# The triadic relationship between spinal posture, loading, and degeneration

**DOI:** 10.3389/fbioe.2025.1444540

**Published:** 2025-03-18

**Authors:** Marie-Rosa Fasser, Pascal R. Furrer, Luca Fisler, Lukas Urbanschitz, Jess G. Snedeker, Mazda Farshad, Jonas Widmer

**Affiliations:** ^1^ Spine Biomechanics, Balgrist University Hospital, University of Zurich, Zurich, Switzerland; ^2^ Institute for Biomechanics, ETH Zurich, Zurich, Switzerland; ^3^ Department of Orthopedics, Balgrist University Hospital, University of Zurich, Zurich, Switzerland; ^4^ Department of Spine Surgery, University Hospital of Basel, Basel, Switzerland

**Keywords:** spinopelvic alignment, spine degeneration, spine biomechanics, musculoskeletal modeling, spine loading

## Abstract

**Introduction:**

Degenerative changes in the lumbar spine may affect many structures, among them the intervertebral discs and the facet joints. The individual load distribution within the spine linked to posture and mass distribution is a probable cause of disease. This study had a dual aim: first, to systematically summarize previously reported associations between sagittal balance parameters and the occurrence of lumbar spine degeneration. Second, to complement these insights with additional biomechanical findings meant to elucidate the link between spine load and alignment as well as selected demographic descriptors.

**Methods:**

A systematic literature search was performed on PubMed to identify clinical studies that quantified the association between spinal alignment and the occurrence of disc herniation, disc degeneration, facet joint degeneration, and spondylolisthesis. Further, a previously published musculoskeletal model was used to link sagittal spinal alignment and subject characteristics to joint loading within the lumbar spine for a cohort of 144 subjects.

**Results:**

The literature review yielded 49 publications evaluating the relationship between spinal alignment and the occurrence of pathologies in the lumbar spine. The results indicate that a straight spine might negatively affect the health status of the intervertebral disc, likely because of a lack of damping and associated high compressive loads. These loads further show a major dependence on body weight. On the other hand, facet degeneration and spondylolisthesis may be linked to higher anterior-posterior shear forces acting on the relevant spinal structures because of a generally more sagittally curved spine. A straight lumbar spine is more likely to stress the disc, whereas highly curved spines with a high pelvic incidence are more likely to stress the posterior structures. The biggest influencing factors on the resulting force and consequently potentially the wear of the anatomical structures are the intervertebral inclination from an anatomical point of view and the weight from a demographic point of view.

**Discussion:**

Information concerning spinal loading resulting from spinal alignment and body descriptors could impact both conservative treatment and operative planning for patients afflicted by spine disease through targeted changes in posture.

## Introduction

Because of its known clinical relevance, the meticulous study of spine biomechanics goes far back in history (Sanan and Rengachary, 1996). Over the years, many fundamental insights regarding the alignment and balance of the spinal and pelvic structures were obtained (Duval-Beaupère et al., 1992; Legaye et al., 1998). An important agreed-upon aspect is the existence of a link between patterns of spinopelvic arrangement and the prevalence of degenerative changes in the spine. Importantly, associations were observed between the degree of sagittal parameters like pelvic incidence (PI) and sacral slope (SS) and facet joint integrity, spondylolisthesis, and pathological changes to the disc (Rajnics et al., 2002; Endo et al., 2010; Yang et al., 2014; Bae et al., 2016; Fei et al., 2017; Wang et al., 2017; Wu et al., 2019; Barrey et al., 2007a; Wu et al., 2020; Oh and Eun, 2015; Menezes-Reis et al., 2016; Ogon et al., 2020; Zehra et al., 2020; Pourabbas Tahvildari et al., 2021; Sahin et al., 2015; Jentzsch et al., 2013; Lv et al., 2016; Labelle et al., 2004; Vialle et al., 2007; Schuller et al., 2011; Funao et al., 2012; Lim and Kim, 2013; 2014; Liu et al., 2015; Oh et al., 2013; Yin et al., 2016; Ferrero et al., 2015; Wang et al., 2016; Chuang et al., 2018; Chuang et al., 2021; Lai et al., 2018; Nakamae et al., 2019; Ha et al., 2019; Barrey et al., 2007b; Bao et al., 2024; Soydan et al., 2023b; Song et al., 2022; Savarese et al., 2022; Muellner et al., 2022a; Muellner et al., 2022b; Shi et al., 2024; Soydan et al., 2023a; Aboushaala et al., 2024; Meng et al., 2024; Kong et al., 2023; Leng et al., 2023; Oyekan et al., 2023; Shrestha et al., 2021; Shi et al., 2022). Despite the availability of many clinical reports about posture-pathology links, they have yet to be summarized systematically for the detection of clear trends and to potentially better understand cause and effect.

The likely biomechanical principles and force distribution patterns predisposing patients to accelerated degeneration have not yet been comprehensively characterized. Because of ethical concerns and technical challenges related to *in vivo* studies, detailed research on such principles may be carried out with computational simulations. Computer-based approaches like musculoskeletal (MSK) modeling are well-established techniques in biomechanics due to their noninvasive character, the quantitative nature of the outcome they provide, and the relative ease with which the isolated effect of parameter variations can be assessed in a well-controlled environment (Christophy et al., 2012; Bruno et al., 2015; Senteler et al., 2014; Senteler et al., 2016; Senteler et al., 2018). Even though they only convey a simplified representation of reality, *in silico* simulations can take into account patient-specific aspects retrieved from imaging data (such as anatomy, spinal alignment, muscle morphology, and mass distribution) (Bruno et al., 2017; Senteler et al., 2017; Caprara et al., 2020; Fasser et al., 2021). Spinal MSK simulations provide insights concerning the neuromuscular activity within the spine by predicting the activity of the muscle fascicles and the loads acting on the joints during static upper body posture or dynamic movement. So far, few computational studies have tried to establish a relationship between sagittal spine alignment and joint loading (Bassani et al., 2019; Müller et al., 2021). However, large cohorts of subjects spanning a broad range of alignment and demographic characteristics required to establish a relationship between biomechanics and alignment that can be linked to clinical outcomes have not yet been analyzed.

We hypothesize that the accelerated occurrence of degeneration is a result of higher forces and therefore wear of the specific anatomical structures, which is linked to the spinopelvic parameters. Therefore, this study aimed to use previously published clinical observations to highlight the trends in correlations between lumbar spinal pathologies and spinopelvic arrangement and to combine the findings with the independently gathered insights on the connection between posture and body descriptors, and joint loading. As a result, a contribution to a better and more holistic understanding of the triadic relationship between spine degeneration, alignment, and loading shall be made. Consequently, we intend to provide rules of thumb for clinicians to better assess the spine loading resulting from individual spinal alignment or demographic descriptors. Such information could impact both conservative treatment and operative planning for patients afflicted by spine disease through targeted changes in spine posture.

## Materials and methods

The project comprised two parts: a systematic literature review and an analysis of biomechanical simulations. The purpose of the literature review was to use available clinical data to investigate the link between posture and the prevalence of pathologies affecting the uninstrumented lumbar spine (Section 2.1) and to comprehensively depict the distinguishable trends. On the other hand, a computational study involving subject-specific simulations for a large cohort was conducted with the main goal of elucidating the relationship between posture and joint loading (Section 2.2). Eventually, the likely relationship between the occurrence of pathology and the forces acting on the lower spine was derived.

### Systematic literature review

**FIGURE 1:**
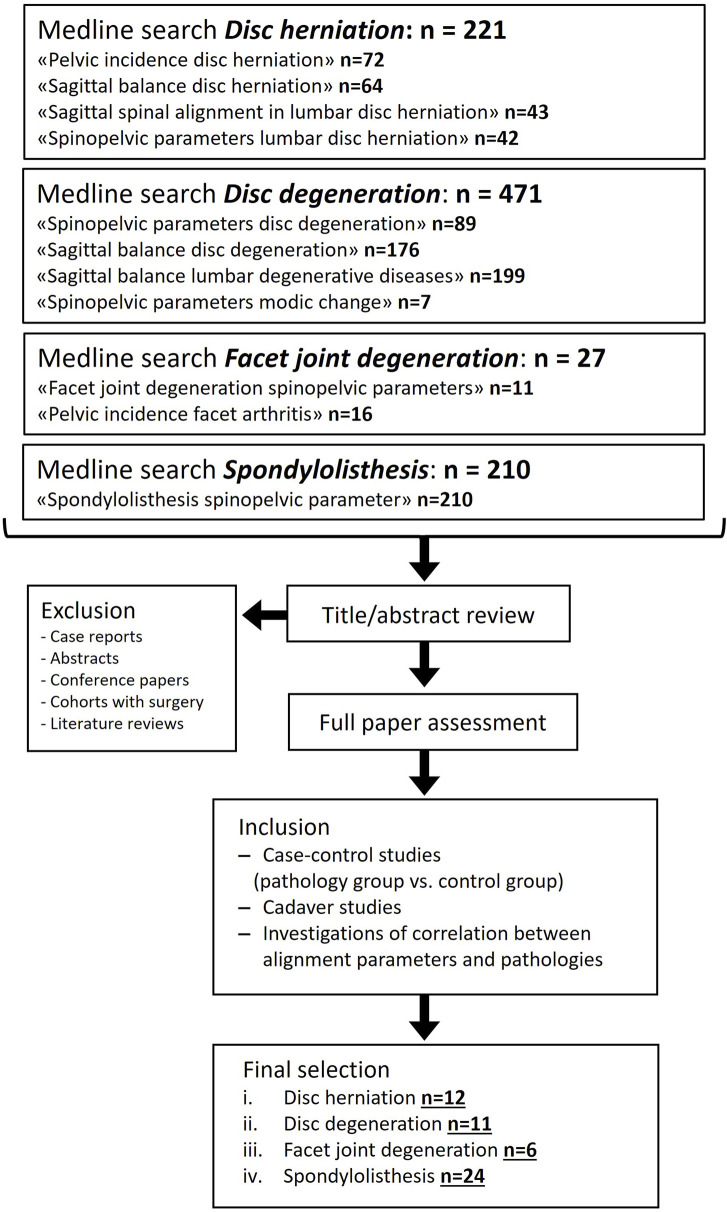
Flow diagram depicting the systematic selection process for study inclusion in the literature review. PubMed search was conducted in December 2024.

For the literature review, “PubMed”^1^ was searched with eleven terms describing four different pathologies affecting the spine (Figure 1). First, abstract screening and data collection were carried out and no automated tools were used. Next, a thorough selection of eligible studies was performed by analyzing full texts. The main inclusion criterium was an available quantitative comparison of spinopelvic parameters between a cohort of people with a spine pathology and an unafflicted control group. Excluded were case reports, abstracts, conference presentations, reviewer comments, review articles, papers focusing on the cervical spine, papers not written in English or German, and duplicates. Studies analyzing spinopelvic parameters in postoperative situations were also excluded. The exclusion criteria were defined in such a way as to find original articles with a meaningful cohort size of non-surgically treated subjects that provided enough information on methodology to ensure a certain level of comparability between the included studies.

### Computational simulations

**FIGURE 2:**
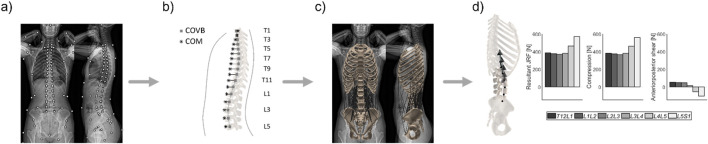
Workflow for the generation of personalized musculoskeletal models. **(A)** Anatomical landmarks were annotated on bi-planar radiographs (EOS imaging, Paris, France). **(B)** The individualized distribution of mass within the upper body and the alignment of the spine were obtained from the annotated images. **(C)** A musculoskeletal model consisting of seven rigid bodies linked by spherical joints and connected by 230 individual muscle fascicles was generated and rendered the anatomy and alignment of the subject in the standing position. **(D)** Optimization based on the assumption of energy efficiency in muscle activity provided information on load distribution within the lower spine during standing. COVB: Center of the vertebral body; COM: Center of mass.

144 subject-specific MSK models were generated within the scope of a previous study (Fasser et al., 2021) and the procedure of model generation is summarized in Figure 2. Demographic data (age, weight, height), postural parameters, and joint loads were recorded and computed. Subjects with scoliotic deformities (i.e., thoracolumbar Cobb’s angle ≥10°) and/or a previous instrumentation surgery in the spine were excluded from the cohort to avoid the potential distortive effect of these conditions on loading and posture (Cobb, 1948). Further information regarding the health status of the subjects was not available.

The sagittal spinopelvic parameters were obtained from the annotated images (Figure 2A) and defined as follows: the SS, the PI, and the pelvic tilt (PT) were computed based on the sagittal orientation of the sacral endplate and the position of the femoral heads (Legaye et al., 1998); lumbar lordosis (LL) and thoracic kyphosis (TK) were characterized as the angles between the superior endplate of L1 and the sacral endplate, and the sagittal angle between the superior endplate of T5 and the inferior endplate of T12, respectively; the sagittal vertical axis (SVA) was described as the horizontal (anterior-posterior) distance between the C7 plumbline and the posterior corner of the sacral endplate. The spinopelvic alignment parameters of the cohort are tabularized in Supplementary Table 1 within the Supplementary Material. Finally, the sagittal intervertebral inclination (IVI) describes the average between the upper and the lower endplate angle of each intervertebral disc (Supplementary Table 1).

The weight and height of the subjects were not available from clinical records. However, weight information was needed to render the individualized mass distribution in the MSK models and was therefore predicted based on the body outline and experimentally derived values for level-dependent density and percentage mass contribution (Pearsall et al., 1996; Fasser et al., 2021). We previously showed that this approach provides satisfactory approximations of the subject’s measured weight (Fasser et al., 2021) (Supplementary Figure 1A). Further, in the bi-planar radiographs part of the head was not depicted and therefore the subjects’ height was not readily available. The gonion (i.e., the apex of the mandibular angle), however, can be readily distinguished and annotated on the radiographs. According to published experimental data, this point lies at about 87% of body height (Chambers et al., 2011). Knowing the length from feet to gonion, the total body height can be predicted. This procedure for height estimation was tested with a dataset comprising 82 subjects with known height and available bi-planar radiographs (mean measured height being 1.67 m, ranging from 1.43 m to 1.90 m; unpublished data). The correlation between measured and predicted body height was *ρ*_*Pearson*_ = 0.94 (p-value < 0.0001). The root mean square error (RMSE) and the mean absolute percentage error (MAPE) were determined to further assess the agreement between predicted values and measured ground truth. The RMSE was 3 cm and the mean absolute percentage error (MAPE) was 1.5% (Supplementary Figure 1B). The predicted weight and height were further used to compute the body mass index (BMI). Additional details concerning the subject cohort and image annotation can be found in Fasser et al., 2021.

Musculoskeletal models based on subject anatomy, alignment, and mass distribution were automatically generated and simulated (Figures 2B, C). Through optimization of muscle activity, the joint loads acting upon the lumbar spine were computed for a neutral standing position (Figure 2D).

### Data analysis

**FIGURE 3:**
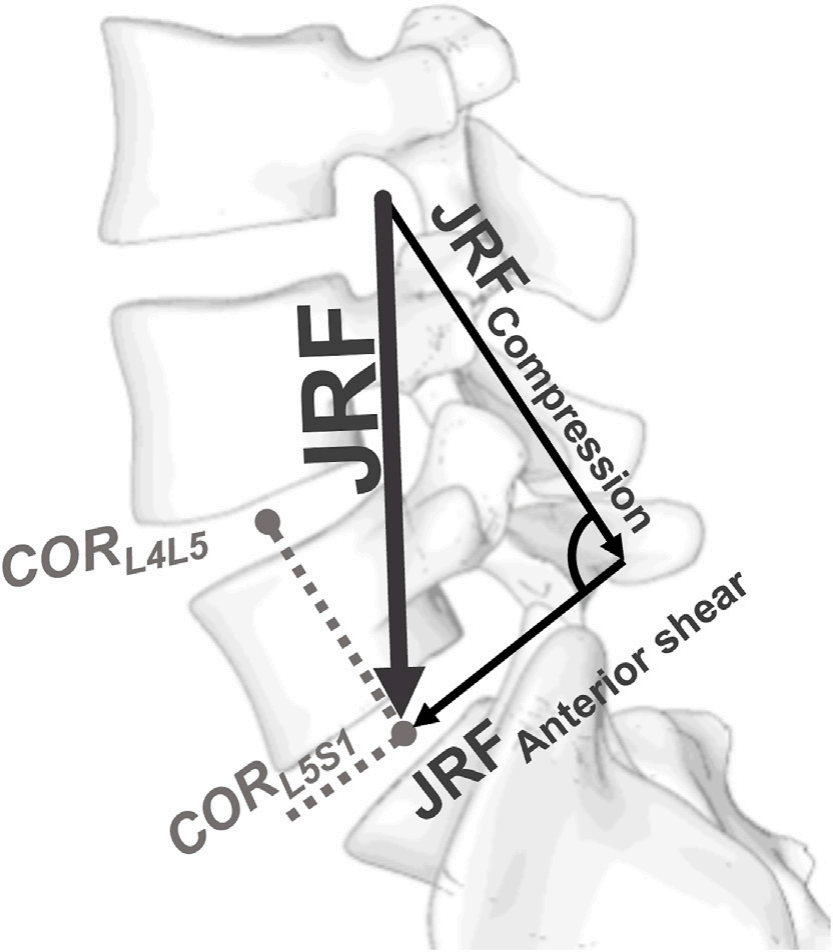
Definition of the local coordinate system at the intervertebral joints. The direction of compression was defined by the vector running from the center of rotation (COR) of the considered segment to the COR of the segment above. The anterior shear direction was perpendicular to the compression direction within the sagittal plane.

The analysis of the quantitative results from the musculoskeletal modeling was performed in MATLAB (R2020b, The MathWorks Inc., Natick, MA, USA) and R (R Core Team, 2022). The resultant joint reaction force (JRF) was decomposed into a compressive and an anterior shear component (Figure 3). To assess the predictability of the JRF, linear regression modeling and an exhaustive parameter search with Bayesian information criterion (BIC) were performed. Linear models were fitted to the data to evaluate the effect of the single spinal alignment parameters/demographic data on the computed joint loads. The Pearson correlation coefficient *ρ* was used to quantify the strength of the association. The level of significance was set to *α* = 0.05.

## Results

### Systematic literature review

**FIGURE 4:**
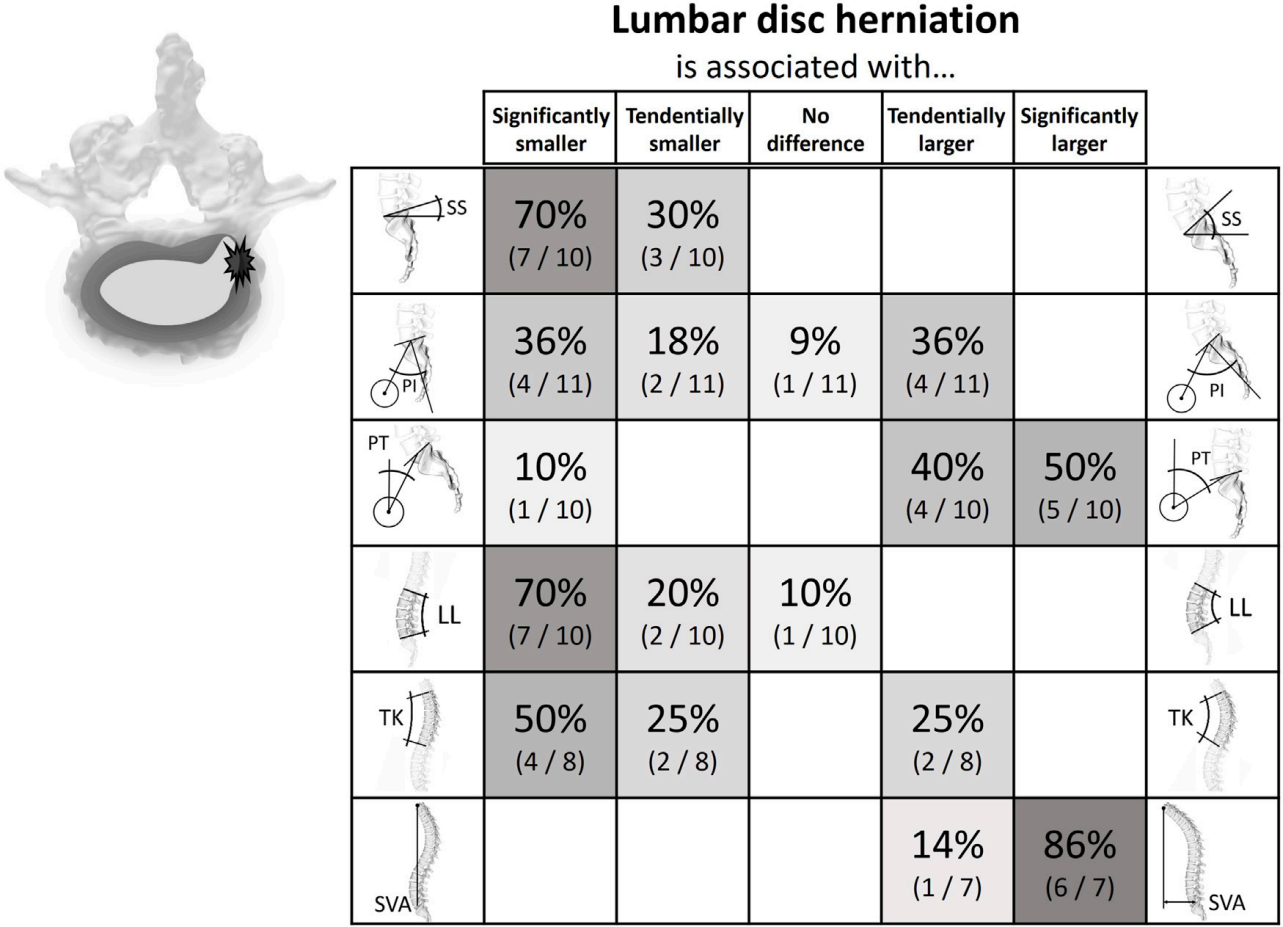
Literature findings on the relationship between spinopelvic alignment parameters and lumbar disc herniation. For example, 70% of the publications comparing the sacral slope between cohorts with lumbar disc herniation and cohorts without the pathology found that the slope is significantly smaller in the case group, while the remainder (30%) reported a non-significant trend in the same direction. Also, 86% of the relevant studies determined a significantly larger sagittal vertebral axis for people with disc herniation.

**FIGURE 5:**
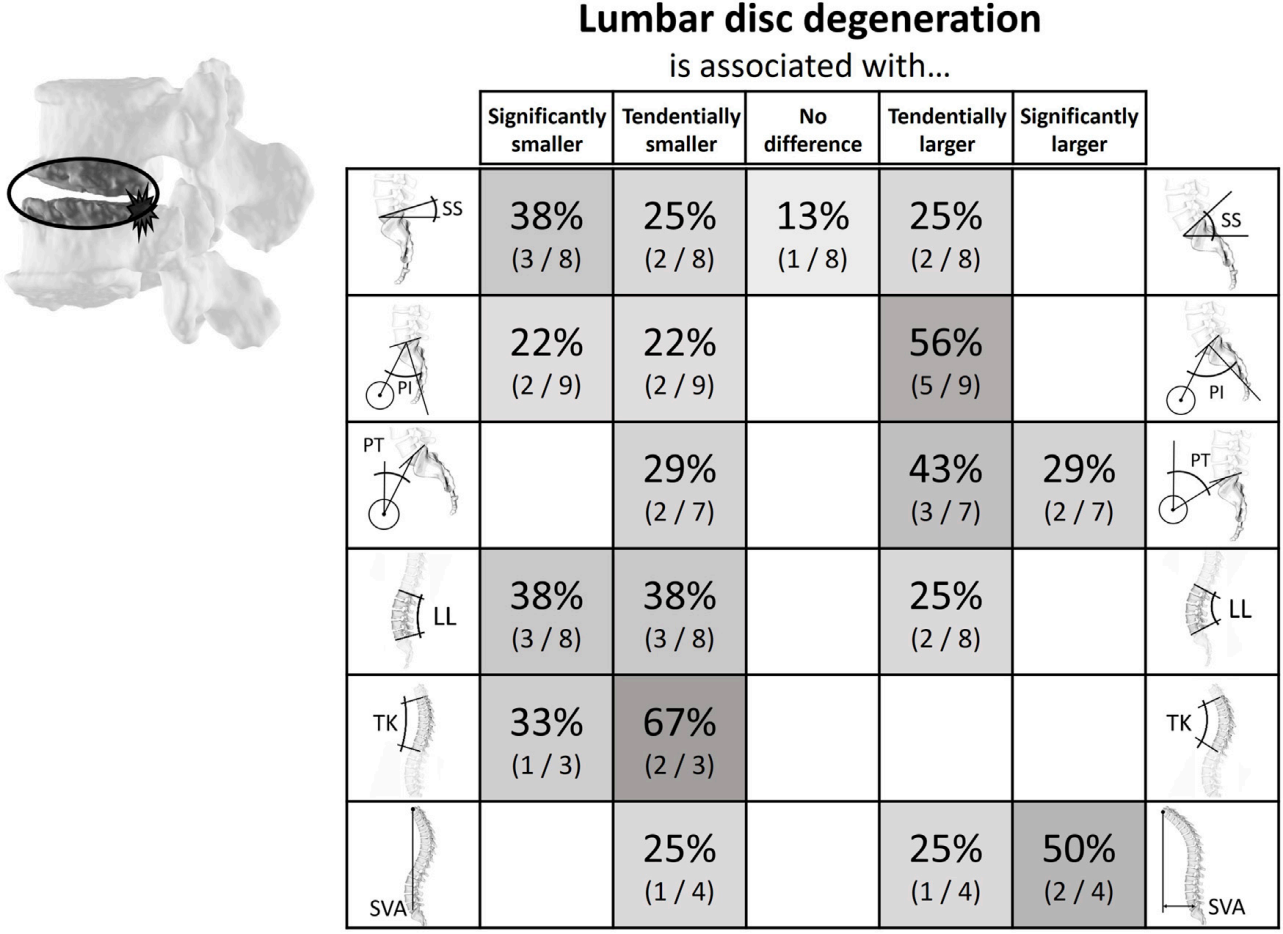
Literature findings on the relationship between spinopelvic alignment parameters and lumbar disc degeneration. For example, 71% of the relevant studies determined the pelvic tilt to be smaller in patients with disc degeneration. Most studies (75%) showed either a tendency for or a significantly smaller lumbar lordosis in the case groups.

**FIGURE 6:**
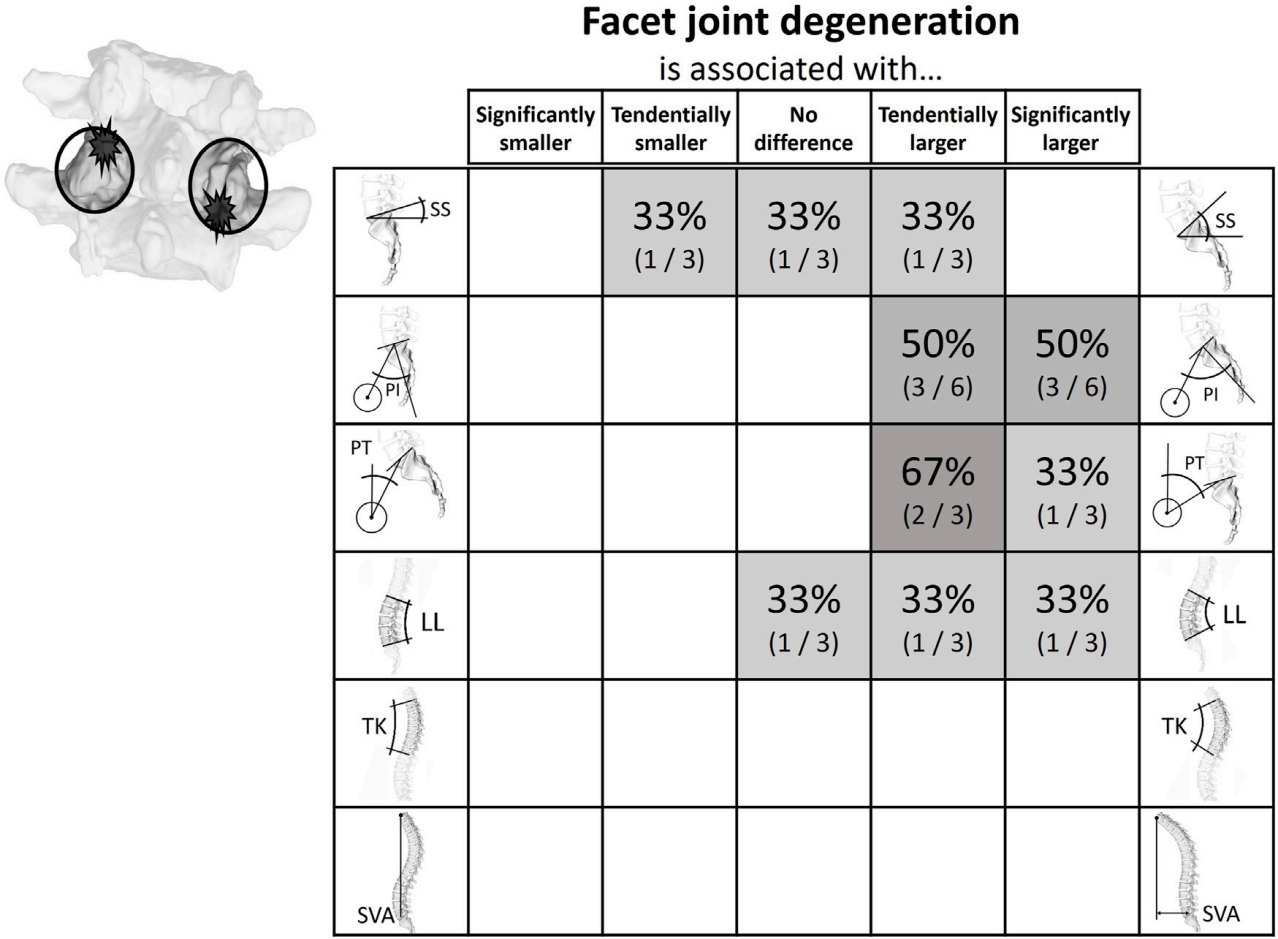
Literature findings on the relationship between spinopelvic alignment parameters and facet joint degeneration. While there did not seem to be a clear indication of an increased or decreased sacral slope, all studies looking at the respective parameters indicated that both the pelvic incidence and the pelvic tilt tended to be larger in the presence of degenerated facet joints.

**FIGURE 7:**
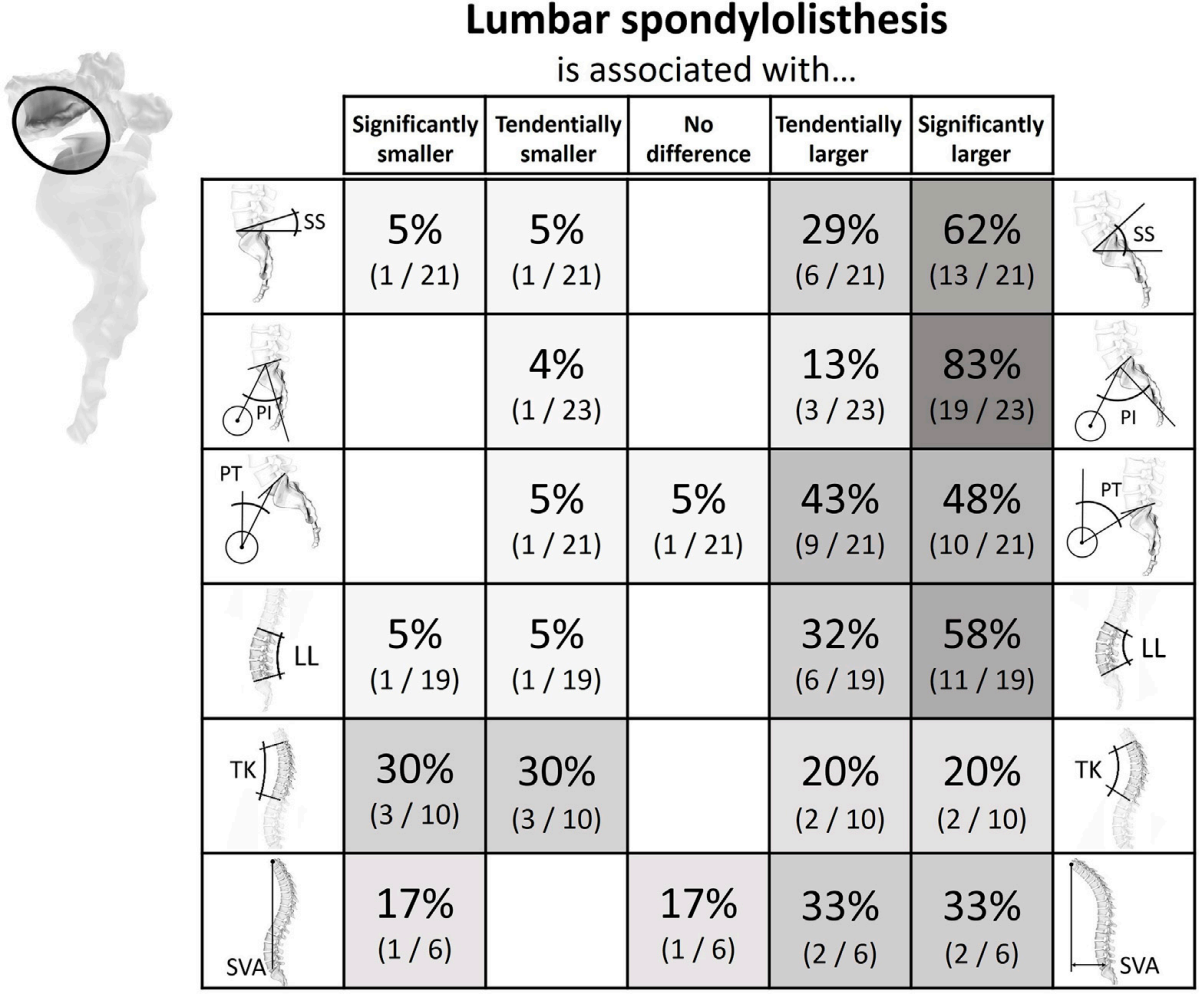
Literature findings on the relationship between spinopelvic alignment parameters and spondylolisthesis. 62% of the studies evaluating the sacral slope in patients with spondylolisthesis detected a significantly larger sacral slope in patients with the condition. As many as 83% of publications reported a greater pelvic incidence in patients compared to unaffected controls. A similar but not equally distinct trend was seen for pelvic tilt and lumbar lordosis. For the sagittal vertical axis, two-thirds of the publications of relevance observed larger values in the case group.

A total of 49 studies were included in the literature review, all of them quantifying the relationship between the posture of the spinopelvic system and the prevalence of certain degenerative conditions affecting the discs and the facets in the lower spine. The literature findings are summarized in Figures 4–7 (Supplementary Figures 2–5): each row corresponds to a different spinopelvic parameter and the strength and directionality of the association vary from left to right. In each box, the percentage of studies in which the respective degree of association was observed, is reported. The boxes of the column in the center (“No difference”) include publications where no numerical differences were reported between the case and control groups. The boxes left and right from the ones in the middle indicate a non-significant tendency towards a smaller or a larger posture parameter amongst the patients with pathological changes in the lumbar spine, respectively.

#### Lumbar disc herniation

Twelve studies analyzed the connection between spinal parameters and lumbar disc herniation (Rajnics et al., 2002; Barrey et al., 2007a; Endo et al., 2010; Yang et al., 2014; Bae et al., 2016; Fei et al., 2017; Wang et al., 2017; Wu et al., 2019; Pourabbas Tahvildari et al., 2021; Song et al., 2022; Soydan et al., 2023b; Bao et al., 2024). Subjects with low LL, SS, and PI showed a tendentially increased rate of occurrence of lumbar disc herniations. The opposite was true for the SVA and the PT, where herniation was linked to higher values (Supplementary Figure 2; Figure 4).

#### Lumbar disc degeneration

Eleven papers comparing spinopelvic parameters between subjects with and without lumbar disc degeneration were found (Supplementary Figure 3; Figure 5). Of these publications seven were cross-sectional and four were case-control studies (Barrey et al., 2007a; Yang et al., 2014; Oh and Eun, 2015; Menezes-Reis et al., 2016; Ogon et al., 2020; Wu et al., 2020; Zehra et al., 2020; Muellner et al., 2022a; Muellner et al., 2022b; Savarese et al., 2022; Soydan et al., 2023b). Seven studies used Pfirrman (Pfirrmann et al., 2001) or MC (Modic et al., 1988) grading for degeneration classification (Yang et al., 2014; Oh and Eun, 2015; Menezes-Reis et al., 2016; Wu et al., 2020; Zehra et al., 2020; Muellner et al., 2022a; Soydan et al., 2023b). Three studies used different categorizations of degeneration (Ogon et al., 2020; Muellner et al., 2022b; Savarese et al., 2022) and one did not specify the degree of degeneration (Barrey et al., 2007a). In general, a correlation between low SS and LL and the occurrence of disc degeneration was described. The opposite was shown for PT, which was tendentially increased in patients with lumbar disc degeneration.

#### Facet joint degeneration

The relationship between spinopelvic parameters and facet joint degeneration (FJD) was assessed by six studies (Supplementary Figure 4; Figure 6). Five of these were cross-sectional studies and one was a case-control study (Jentzsch et al., 2013; Sahin et al., 2015; Lv et al., 2016; Muellner et al., 2022b; Soydan et al., 2023a; Shi et al., 2024). To classify FJD, two studies used the Pathria classification, three the Weisshaupt classification, and one classified facet joints as degenerated in case of joint space narrowing and osteophytes present around the joint (Pathria et al., 1987; Weishaupt et al., 1999). In three studies the spinopelvic parameters were measured using only computed tomography (CT) scans, where only PI was used for the description of the sacropelvic alignment due to the prone positioning (Jentzsch et al., 2013; Sahin et al., 2015; Muellner et al., 2022b). The other three studies used lateral x-ray images acquired during upright standing for the measurement of the spinopelvic parameters and CT or MRI scans for analysis of the FJD (Lv et al., 2016; Soydan et al., 2023a; Shi et al., 2024). All studies showed an association between high PI and PT and an increased incidence of FJD.

#### Spondylolisthesis

In twenty-four publications the correlation between spinopelvic parameters and spondylolisthesis was analyzed (Supplementary Figure 5; Figure 7). The reason for listhesis was spondylolysis in seven studies, degenerative in fifteen investigations, developmental in one study, and both spondylolysis and degenerative in another study (Labelle et al., 2004; Barrey et al., 2007b; Vialle et al., 2007; Schuller et al., 2011; Funao et al., 2012; Lim and Kim, 2013; Lim and Kim, 2014; Oh et al., 2013; Ferrero et al., 2015; Liu et al., 2015; Wang et al., 2016; Yin et al., 2016; Chuang et al., 2018; Chuang et al., 2021; Lai et al., 2018; Ha et al., 2019; Nakamae et al., 2019; Shrestha et al., 2021; Shi et al., 2022; Kong et al., 2023; Leng et al., 2023; Oyekan et al., 2023; Aboushaala et al., 2024; Meng et al., 2024). Two studies were conducted with a cross-sectional design, and the remainder were case-control studies. The vast majority of investigations could show that SS, PI, PT, and LL are elevated in patients with spondylolisthesis. Further, one study showed that the listhesis group additionally had an increased BMI (Schuller et al., 2011), and another showed that the degree of listhesis in patients with spondylolysis also increased with increasing discus degeneration and age (Chuang et al., 2021).

### Computational simulations

The considered alignment and demographic parameters were highly correlated to each other and the prediction of JRF with models incorporating all parameters (age, weight, height, BMI, TK, LL, SVA, SS, PT, PI, IVIL4L5, and IVIL5S1) would likely lead to extensive overfitting. The compressive forces at the L4L5 joint are best predicted by a linear regression model only taking weight and the IVIL4L5 into account (R_adj_^2^ = 0.88). The compressive forces at the L5S1 joint, on the other hand, can effectively be predicted by a model based on weight, SS, and both IVIL4L5, and IVIL5S1 (R_adj_^2^ = 0.76). Shear forces were less well predictable. Weight, PI, IVIL4L5, and IVIL5S1 lead to the best model for the shear force in L4L5 (R_adj_^2^ = 0.53), and a regression fit based on weight, PT, and IVIL4L5 was possibly the best option for the shear force at the L5S1 level (R_adj_^2^ = 0.69).

#### Posture and load

**FIGURE 8:**
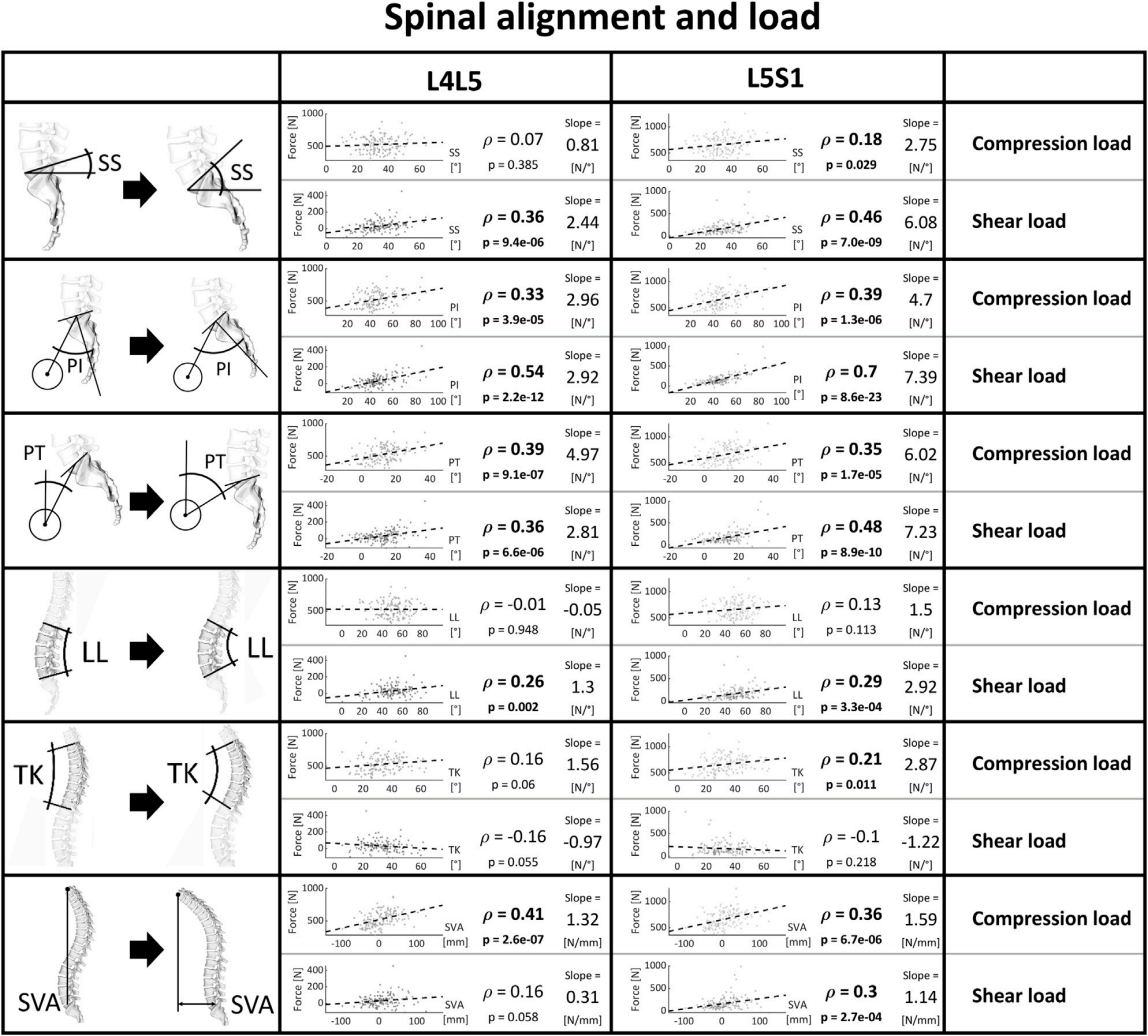
Strength of association between spinopelvic alignment and joint load. Significant correlations are written in bold and the slope refers to the regression line fitted on the data.

Figure 8 depicts the extent of the correlation between parameters describing posture and the loads predicted at the corresponding L4L5 and L5S1 joints with the patient-specific musculoskeletal models. All three sacropelvic parameters and mostly the PI were strongly linked to the shear components of the JRFs. For PI and PT, there was also a clear association with the compressive force components. Further, while an increased SS inclination seems to be accompanied by an increased shear force, there was only a slight increase in compression force. LL was positively linked to an increased anterior shear in the L4S1 region, while TK was weakly linked to the compression in L5S1. In general, the correlation between the JRF and both LL and TK was relatively weak for both compression and shear compared to the strong associations observed for the sacropelvic parameters. An increased SVA was strongly linked to higher compressive force components and larger anterior shear at L5S1.

**FIGURE 9:**
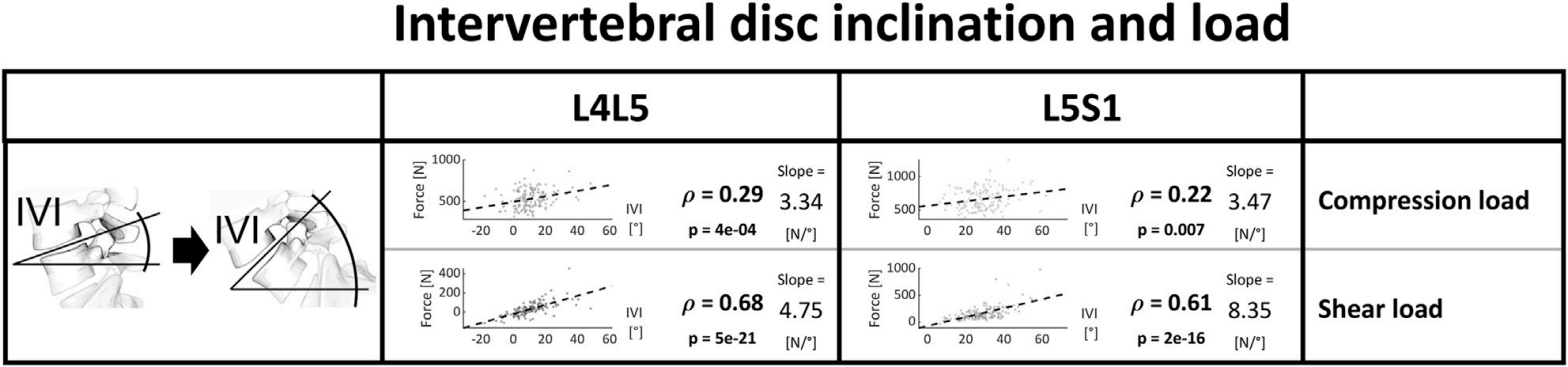
Strength of association between intervertebral inclination and joint load. Significant correlations are written in bold and the slope refers to the regression line fitted on the data.

A more prominent inclination of the intervertebral disc at the lower lumbar level was consistently associated with higher joint reaction forces, with a more considerable link to anterior shear (Figure 9).

#### Demographic parameters and load

**FIGURE 10:**
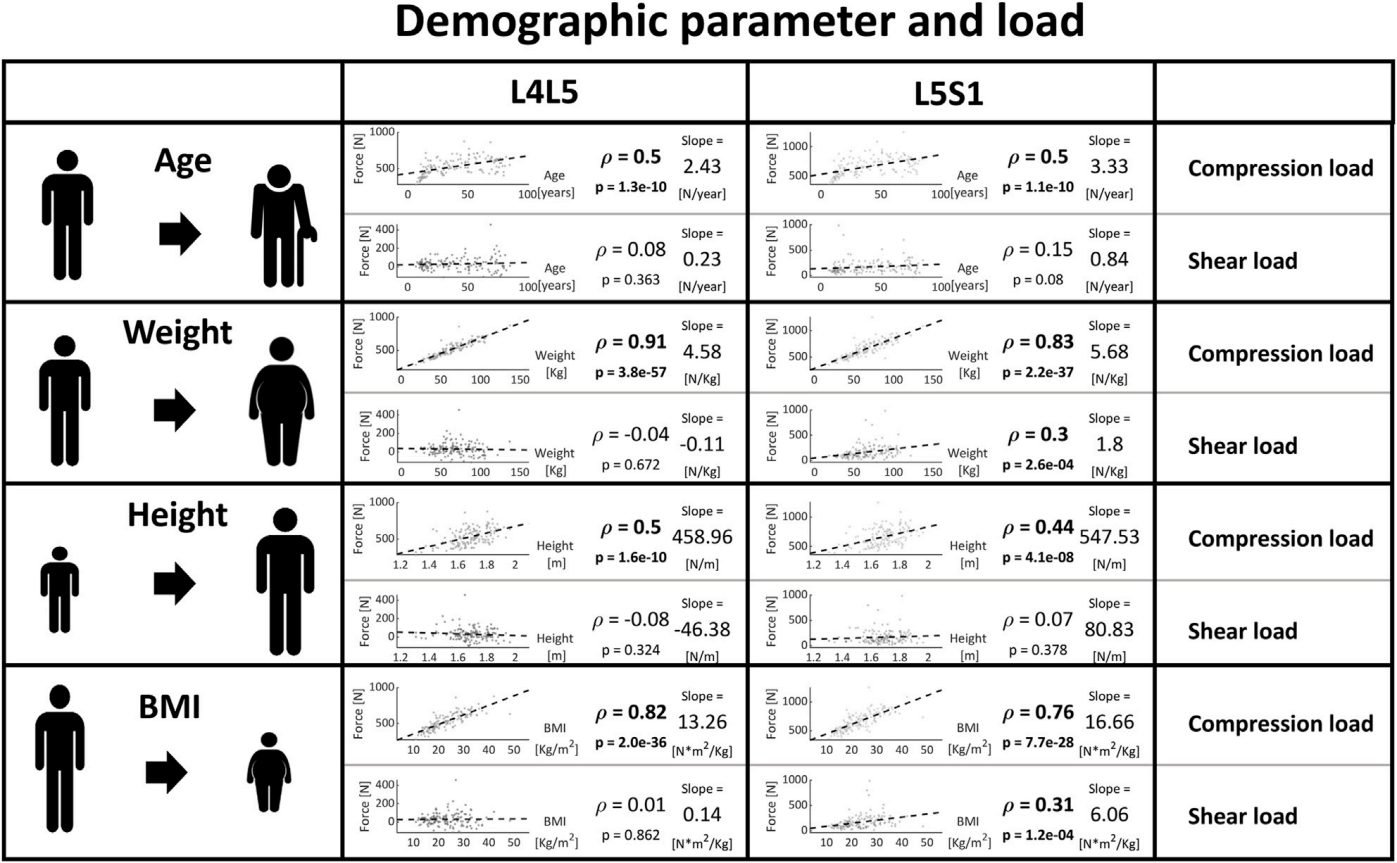
Strength of association between patient characteristics (age, weight, height, BMI) and load at the caudal lumbar joints. Significant correlations are written in bold and the slope refers to the regression line fitted on the data.

Age, weight, height, and BMI were correlated to the segmental forces. An increase in these parameters was strongly linked with an increase in vertebral compression forces, especially for weight (and consequently also BMI). Also, weight and BMI were weakly associated with shear at the L5S1 level and all other combinations of level and demographic parameters were not correlated to shear forces (Figure 10).

## Discussion

The results of the conducted systematic literature review suggest the existence of a clear link between lumbar spinal degeneration and spinopelvic alignment. Computationally predicted loads within the lower spine for a subject cohort that covers a broad range of spine alignments and demographic characteristics provide a possible explanation for the observed trends.

### Systematic literature review

The systematic literature review highlighted patterns of association between spinopelvic parameters and spinal degeneration. Intervertebral disc herniation and degeneration were found to be clustered in patients with a rather straight vertical alignment, i.e., with low SS and low LL (Figures 4, 5). The PT tends to have a less clear but opposite relationship with disc herniation and degeneration. This tendency for high PT could be due to a compensational adaptation of posture to limit the translation of the gravity axis towards anterior (Barrey et al., 2007a; Barrey et al., 2011; Endo et al., 2010). Also, the higher prevalence of disc pathologies observed with an increased SVA could be the sign of an adapted upper body posture for pain relief rather than a direct cause of the degenerative tendency. For example, if the herniated disc impinges nerve roots, leaning forward might lead to a reduction of the consequently arising pain (Inufusa et al., 1996; Hira et al., 2021). This is further supported by the circumstance that this type of sagittal imbalance is usually quickly restored after spondylodesis surgery (Endo et al., 2010). Degeneration of posterior structures such as FJD and spondylolisthesis were mainly found in patients with opposite pelvic configuration, i.e., a more bent spine and, namely, high PI, high SS, and high LL (Figures 6, 7). The tendency for increased pelvic tilt with FJD might seem somewhat counterintuitive. Since retroversion can be a compensatory mechanism following the anterior center of gravity translation, a high pelvic tilt would not necessarily be expected as a posture adaptation in FJD. The tendency for an increased PT in the case of FJD would therefore suggest this is a direct mechanism for facet joint relief rather than a compensatory measure. The lumbar levels most commonly affected by the investigated degenerative pathologies are the more caudal ones, which are exposed to the highest loads (Teraguchi et al., 2014). Hence, there is no clearly and consistently discernible link between TK and the considered pathologies. The observed trends indicate that configurations with more horizontally aligned endplates are likely to be exposed to less damping and higher relative compressive forces. This in turn might induce higher strains on the intervertebral discs, ultimately paving the way for their degeneration. On the other hand, a more curved lumbar spine and highly inclined vertebrae could expose the spinous structures to higher shear loading and lead to the degeneration of facets and spondylolisthesis (Zehra et al., 2020). With increased sacral inclination a greater ventrally pulling shear force exists and with a flatter alignment, the pressure component on the vertebral disc is dominant. However, the shear forces are likely to not be exclusively absorbed by the posterior structures, and the compressive forces are probably not only absorbed via the intervertebral disc. One recent study found that in patients with spondylolysis the grade of listhesis increases with the degeneration of the disc, which suggests a certain level of shear force absorption of the disc (Chuang et al., 2021).

Descriptors of global alignment like the Roussouly classification and the global alignment and proportion (GAP) score have emerged as valuable parameters to characterize global spinal alignment and predict post-operative complications (Roussouly et al., 2005; Yilgor et al., 2017). This study aimed to focus primarily on the isolated effect of more regional parameters (except for the SVA), the combination of which could then be used to describe a more global and complex picture. Eventually, combining insights from global and regional alignment descriptors could provide a more comprehensive perspective of spinal alignment.

The focus of this literature review lies on the spinopelvic alignment parameters and their connection to the prevalence of degenerative pathologies affecting the spine. Although not the primary subject of this research, it is important to position the above-mentioned findings in the context of the changes at the molecular and cellular level which spinous tissues such as the intervertebral disc, the ligaments, and the muscles undergo during aging and degeneration. At the cellular level an unfavorable interplay between the biomechanics, the activity and the differentiation status of the cells, and the composition and structure of the surrounding matrix has been described to be involved in degeneration (Crean et al., 1997; Vergroesen et al., 2015). For example, with aging the expression of proteoglycans, elastic fibers, and the ratio of type II to type I collagen decreases while on a structural level, the extracellular matrix in the disc becomes less organized and dehydrated during degeneration therefore negatively impacting the biomechanical characteristics of the IVD (Fontes et al., 2019; Zeldin et al., 2020; Dou et al., 2021). The resulting changed load transfer within the tissue can then cause and be worsened by annular delamination and tears, which in turn result in tissue degeneration and compensatory spinal alignment changes. On the other hand, mechanical stimuli like hydrostatic pressure and cyclic stretch have been shown to impact cell proliferation, apoptosis, and extracellular matrix homeostasis through signaling pathways involving mechanosensitive proteins like integrins and cadherin. These effects were shown to depend upon the magnitude of loading (Wang D. et al., 2021; Wang et al., 2021 Y.). Finally, also the presence of co-morbidities and genetic predispositions are aspects potentially affecting the degeneration status within the spine.

### Computational simulations

As mentioned in the previous section, the considered degenerative pathologies of the spine mostly affect the levels exposed to the highest loads, i.e., the most caudal ones. Therefore, we only analyzed joint loads at L4L5 and L5S1. Also, the association between spinopelvic parameters and pathologies is most prominent in the lower spine (Lv et al., 2016). In general, the joint reaction forces predicted through the optimization of musculoskeletal systems exhibited considerable correlations with spinopelvic parameters. A clear and intuitive trend for greater shear forces with higher spinopelvic angles was determined. In subjects with comparatively high values for sacropelvic parameters, there was an increased anterior shear, a postural configuration associated in the literature with increased FJD and degenerative spondylolisthesis. Despite not being a parameter usually assessed for determining the sagittal balance, the inclination of the intervertebral disc showed a similar and consistent trend. A possible advantage of considering IVI instead of other sagittal alignment parameters to determine balance and the risk for degeneration is the restricted image field of view necessary to quantify the endplate angles in the lumbar spine. Such an index could be used to reliably assess the force distribution for each segment and could gain importance in the treatment of patients with back pathologies.

Other computational studies have investigated the relationship between spinal alignment and joint loads. Bassani et al. conducted a study where the spinopelvic alignment of a representative European male was parametrically changed in a musculoskeletal model and they saw no association between pelvic incidence and joint forces in L4L5 or L5S1 segment, but there was an impact on the loads acting on those joints from variations in global sagittal alignment and sacral slope (Bassani et al., 2019). Müller et al. recently simulated the loading for 28 subjects without discernable spine degeneration to assess the influence of lumbar lordosis variations on joint loading. While in our study we detected moderate dependencies between the shear component of JRFs and LL, they observed considerable LL-dependent effects (Müller et al., 2021).

As was done for the above-mentioned literature review, in this second part of the investigation we focused on alignment parameters which are mostly characterizing the regional rather than the global spinopelvic alignment. However, the interplay of such local parameters more than their stand-alone effects has been suggested to be of great value (Roussouly et al., 2005; Yilgor et al., 2017). When looking at the link between several descriptors of global alignment (for example, T1 pelvic angle, C7 to sacro-femoral distance ratio, global tilt, as well as the GAP score and the Roussouly classification) and the joint loading within the lower lumbar spine, none of the mentioned parameters showed a stronger association with the load compared to the already reported alignment and demographic aspects (Supplementary Figures 6–10).

Differences in loading between male and female subjects were observed but the results were not included since the reported alignment and demographic parameters are expected to explain most of the effect. Specifically, differences in spine load between female and male subjects are likely to result from variations in alignment, body weight, height, and mass distribution/shape.

The outcome from musculoskeletal modeling also allows us to connect general subject descriptors like weight and height and forces, which could become relevant for operative indications, patient care, and patient information. Most importantly, weight and BMI were highly linked to vertebral compression forces within the joints (Teraguchi et al., 2014). Thus, it becomes evident that for the protection of intervertebral disc health and integrity, the response to the patient’s weight or BMI is of decisive importance, especially in overweight people.

It is possible to draw some general conclusions on the quantitative relationship between spinopelvic alignment and weight and loading of the single lumbar joints, which serve as rules of thumb when assessing the impact of variable changes. These may allow clinicians to obtain a better understanding of how intraoperative or perioperative modifications in sacropelvic alignment as well as weight changes can increase the loading of the spine and therefore potentially affect the risk for degenerative changes:

• Sacropelvic orientation: the shear force increases by 20–30 N (2–3 Kg) and 60–70 N (6–7 Kg) for every additional 10° at the L4L5 joint and L5S1 joint, respectively.
• The inclination of the intervertebral space: the compressive and the anteroposterior shear components of the forces increase by 30 N (3 Kg) and 50–80 N (5–8 Kg) per 10 degrees of sagittal intervertebral inclination relative to the horizontal plane, respectively.
• Weight: for each additional kg of body weight, each segment of the lumbar spine is subjected to roughly 5 N (0.5 Kg) more compressive load.

## Limitations

Some limitations associated with the literature search should be mentioned. First, the levels affected by the various pathologies were not consistently reported. Instead of a level-wise analysis, the overall prevalence of the degeneration within the lumbar spine was presented. This was done to facilitate the interpretation of the results and because of the lack of consistent reporting in the literature. For the same reasons, we did not distinguish between the aetiologies of the pathologies (for example, between isthmic and degenerative spondylolisthesis). Further, age and weight have been demonstrated to correlate with the development of degenerative pathologies affecting the spine (Teraguchi et al., 2014). For some combination of parameters and pathologies, few or no studies were available. Therefore, those results should be interpreted with care. The subjects in the cohorts included in the considered studies differed substantially from each other and stratification of the results based on sex, age, and weight was lacking in our analysis. However, for example, in the case of spondylolisthesis, the literature review cohorts which included younger subjects did not seem to show different trends than the studies which included cohorts with considerably higher average age (Labelle et al., 2004; Oh et al., 2013). Further, functional outcomes and level of evidence of the single studies were not reported. Next, LL, PT, and also SS can be influenced by the patient’s momentary posture during image acquisition. However, considering the high consistency of the reported literature, it is unlikely that this has a major influence on the reported results. Finally, we are only able to make assumptions about the underlying causal chain, the insights that emerged from the literature review are merely associations between alignment and pathologies (Barrey et al., 2011). We did not explicitly evaluate if changes in alignment and posture can potentially relieve symptoms. To make reliable statements about which postural aspects are likely causes of degeneration and which are more likely to be the consequence of such degeneration due to behavioral adaptations induced by pain and discomfort, a prospective longitudinal clinical study investigating the spinopelvic alignment and its health-status-dependent alterations over time would be required. Osteoporotic vertebral fractures and their association with spinopelvic alignment and demographic parameters have not been considered in the present investigation due primarily to the potential bias introduced by systemic low bone quality. Future investigations should look at the effect of bone fractures on (progressive) alignment changes and their dependency on spinal loading.

There were also some limitations related to the computational analysis. Common simplifications were implemented, such as the lack of passive soft tissue contributions and the non-subject-specificity of the muscle properties. Nevertheless, we do not expect these aspects to have a major impact on force distribution prediction during standing (Bruno et al., 2017; Widmer et al., 2020). Further, the sagittal alignment parameters were not directly measured from the biplanar radiographs, but instead computed based on the labels on the scans. Also, muscle integrity was assumed in our models. However, the size and integrity of postural muscles can be linked to degeneration as well. For example, it has been suggested that the cross-sectional area of the psoas major muscle is reduced at the level and side of disc herniation (Dangaria and Naesh, 1998) and it was reported that the sagittal alignment in patients with LDH might be affected by tonic contraction of the lumbar paraspinal muscles (Endo et al., 2010). Also in this case the question about causality, i.e., if muscle atrophy causes degeneration or if lower muscle cross-section is the consequence of degeneration, can not be easily answered (Xia et al., 2019). (Compressive) loads predicted during standing might be limited in relevance for herniation development (Adams and Hutton, 1985; Wilke et al., 2013). The complex interplay between shear, bending and the rate of load application are likely to play a major role in the generation of disc damage in the healthy disc and herniation of the nucleus pulposus after a defect in the annulus fibrosus develops (Wade et al., 2014; Wade, 2017; Schollum et al., 2018; Zengerle et al., 2021).

## Conclusion

Although caution in drawing conclusions is called for, the insights from clinical research and from computer modeling approaches could be combined to better understand the observations obtained from the first by making use of the latter and ultimately unravel the supposedly intricate relationship between spine loading and pathology.

The literature review demonstrated the existence of a clear relationship between the spinopelvic parameters and lumbar degeneration. Although it has not been established which of the two aspects is the cause and which is the consequence as well as what role changed muscle activation plays in this, a low lumbar lordosis might be more likely to stress the disc in the axial direction, whereas highly curved spines paired with a high PI may be more likely to stress the posterior structures and the disc along the anterior-posterior direction. In terms of biomechanical findings, a strong correlation between sacropelvic parameters and shear forces within the lower lumbar levels was observed, therefore consolidating the evidence that increased anterior shear could be linked to the degeneration of facets and the development of listhesis. Further, since the compressive force is strongly linked to a subject’s weight and shear forces are associated with IVI, the joint load is likely to be well predicted based merely on body weight and the inclination of the intervertebral disc in the sagittal plane.

Targeted changes in the spatial position of the pelvis might help in conservative treatment and spine alignment can be addressed specifically in fusion surgery. Further, it might become possible to assess the degree of postoperative loading of adjacent segments to better estimate sustainability or prognosis.

## Outlook

Next, we will assess the lumbar disc health status of the subjects included in the cohort for computational modeling and assess if there is a connection with the predicted force components. This shall further improve the understanding of the triadic relationship between posture, joint load, and pathology.

## Data Availability

The datasets presented in this article are not readily available because they are patient data. Requests to access the datasets should be directed to JW, jonas.widmer@hest.ethz.ch.

## Footnotes

1
https://www.ncbi.nlm.nih.gov/pubmed/, last accessed: January, 2025
