# Supplementary material for: The triadic relationship between spinal posture, loading, and degeneration
Source: Front Bioeng Biotechnol. 2025 Mar 18;13:1444540. doi: 10.3389/fbioe.2025.1444540 (PMC11959076; doi:10.3389/fbioe.2025.1444540)
Supplement: Supplementary file 1 [file DataSheet1.pdf]

Supplementary Material

	Mean	Standard deviation	Range (min-max)
Age [years]	39	24	7 - 85
Weight [Kg]	66.6	22.8	18.8 - 137.0
Height [m]	1.69	0.12	1.31 - 1.96
BMI [Kg/m^2]	23.2	7.1	11.0 - 48.4
Sacral slope [˚]	34.4	10.2	10.4 - 64.1
Pelvic incidence [˚]	46.9	12.9	15.4 - 89.2
Pelvic tilt [˚]	12.5	9.1	-11.1 - 37.6
Lumbar lordosis [˚]	50.3	13.7	0.8 - 83.2
Thoracic kyphosis [˚]	33.1	11.4	5.5 - 63.6
Sagittal vertical axis [mm]	6.6	35.6	-96.4 - 124.6
T12L1 IVD orientation [˚]	-17.5	7.6	-39.0 - 16.4
L1L2 IVD orientation [˚]	-16.8	7.1	-38.4 - 7.3
L2L3 IVD orientation [˚]	-11.3	7.8	-36.7 - 11.4
L3L4 IVD orientation [˚]	-1.9	8.8	-27.2 - 27.5
L4L5 IVD orientation [˚]	10.6	10.0	-17.7 - 48.1
L5S1 IVD orientation [˚]	29.1	10.0	6.5 - 62.0

Table 1: *Mean, standard deviation, and range (minimum-maximum) of values are specified for demographic and spinopelvic parameters. The dataset consists of 144 (demographic parameters) and 145 (sagittal spinopelvic parameters and disc orientation) subjects for which musculoskeletal models were generated and joint loads in the neutral standing position were computed. Demographic information for one subject was not available for all studied aspects. Adapted from* Fasser et al., 2021.

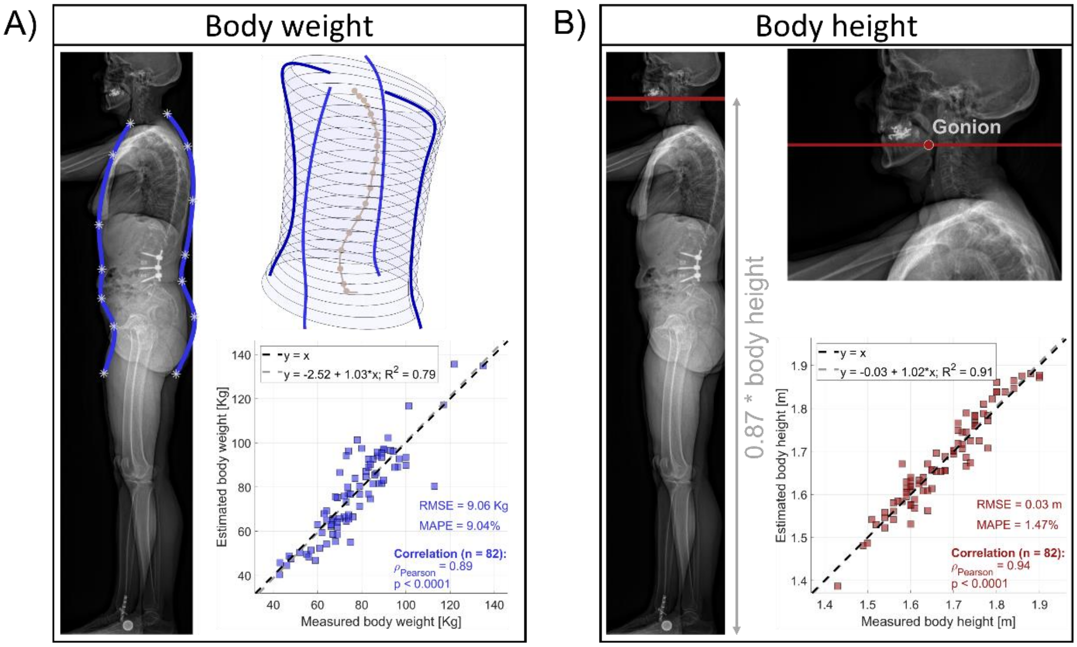

Figure 1: *Prediction of weight and height based on information retrieved from EOS scans and tested with a set of more than 80 EOS images from subjects with known weight and height (unpublished data). These images originated from subjects that were not considered for the simulation part of the main investigation and were only used for validation of the weight and height prediction. The patients had undergone posterior spinal fusion surgery at one or multiple levels between L2 and S1(3xL2L3, 1xL2L4, 3xL2L5, 5xL3L4, 12xL3L5, 4xL3S1, 25xL4L5, 13xL4S1, 16xL5S1) for any reason not related to fracture, infection, non-union, or tumors. The average age of the cohort (36 males, 46 females) was 63 years (standard deviation: 14 years) and the average BMI was 27.4 Kg/m^2 (standard deviation: 4.8 Kg/m^2). A) To predict the weight, the outline of the upper body was determined by the fitting of a spline through annotations on both the sagittal and the frontal EOS scans. The resulting four splines (anterior, posterior, left, and right outline) allowed for rendering the coarse 3D volume of a torso by fitting ellipses in the transversal planes. The level-dependent density and percentage mass contribution values necessary to compute the body weight based on volume were obtained from previously published experimental data (for more details, please refer to* Fasser et al., 2021*)* (Pearsall et al., 1996). *B) Through annotation of the gonion on the sagittal EOS scan and by knowing from experimental measurements at which percentage of body height the gonion usually lies, the full body height was determined* (Chambers et al., 2011).

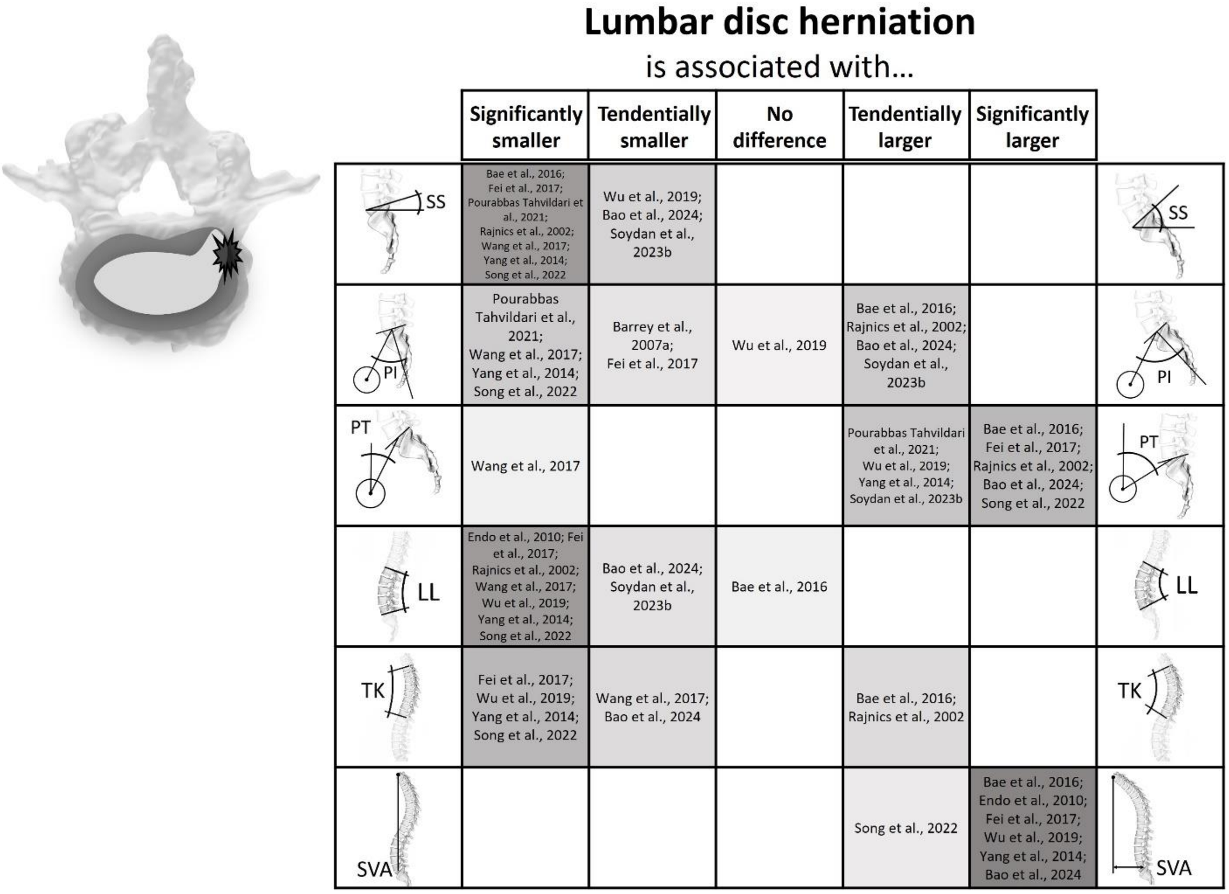

	Significantly smaller	Tendentially smaller	No difference	Tendentially larger	Significantly larger	
SS	Bae et al., 2016; Fei et al., 2017; Pourabbas Tahvildari et al., 2021; Rajnics et al., 2002; Wang et al., 2017; Yang et al., 2014; Song et al., 2022	Wu et al., 2019; Bao et al., 2024; Soydan et al., 2023b				SS
PI	Pourabbas Tahvildari et al., 2021; Wang et al., 2017; Yang et al., 2014; Song et al., 2022	Barrey et al., 2007a; Fei et al., 2017	Wu et al., 2019	Bae et al., 2016; Rajnics et al., 2002; Bao et al., 2024; Soydan et al., 2023b		PI
PT	Wang et al., 2017			Pourabbas Tahvildari et al., 2021; Wu et al., 2019; Yang et al., 2014; Soydan et al., 2023b	Bae et al., 2016; Fei et al., 2017; Rajnics et al., 2002; Bao et al., 2024; Song et al., 2022	PT
LL	Endo et al., 2010; Fei et al., 2017; Rajnics et al., 2002; Wang et al., 2017; Wu et al., 2019; Yang et al., 2014; Song et al., 2022	Bao et al., 2024; Soydan et al., 2023b	Bae et al., 2016			LL
TK	Fei et al., 2017; Wu et al., 2019; Yang et al., 2014; Song et al., 2022	Wang et al., 2017; Bao et al., 2024		Bae et al., 2016; Rajnics et al., 2002		TK
SVA				Song et al., 2022	Bae et al., 2016; Endo et al., 2010; Fei et al., 2017; Wu et al., 2019; Yang et al., 2014; Bao et al., 2024	SVA

Figure 2: Literature findings on the relationship between spinopelvic alignment parameters and lumbar disc herniation (Rajnics et al., 2002; Barrey et al., 2007a; Endo et al., 2010; Yang et al., 2014; Bae et al., 2016; Fei et al., 2017; Wang et al., 2017; Wu et al., 2019; Pourabbas Tahvildari et al., 2021; Song et al., 2022; Soydan et al., 2023b; Bao et al., 2024).

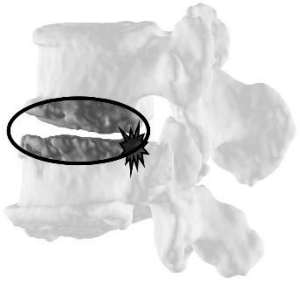

Lumbar disc degeneration

is associated with…

	Significantly smaller	Tendentially smaller	No difference	Tendentially larger	Significantly larger	
SS	Menezes-Reis et al., 2016; Ogon et al., 2020; Yang et al., 2014	Wu et al., 2020; Savarese et al., 2022	Oh and Eun, 2015	Zehra et al., 2020; Soydan et al., 2023b		SS
PI	Wu et al., 2020; Yang et al., 2014	Barrey et al., 2007a; Oh and Eun, 2015		Ogon et al., 2020; Zehra et al., 2020; Soydan et al., 2023b; Savarese et al., 2022; Muellner et al., 2022b		PI
PT		Oh and Eun, 2015; Wu et al., 2020		Yang et al., 2014; Zehra et al., 2020; Soydan et al., 2023b	Ogon et al., 2020; Savarese et al., 2022	PT
LL	Ogon et al., 2020; Wu et al., 2020; Yang et al., 2014	Menezes-Reis et al., 2016; Oh and Eun, 2015; Savarese et al., 2022		Zehra et al., 2020; Soydan et al., 2023b		LL
TK	Yang et al., 2014	Ogon et al., 2020; Savarese et al., 2022				TK
SVA		Savarese et al., 2022		Muellner et al., 2022a	Ogon et al., 2020; Yang et al., 2014	SVA

Figure 3: Literature findings on the relationship between spinopelvic alignment parameters and lumbar disc degeneration (Barrey et al., 2007a; Yang et al., 2014; Oh and Eun, 2015; Menezes-Reis et al., 2016; Ogon et al., 2020; Wu et al., 2020; Zehra et al., 2020; Muellner et al., 2022a, 2022b; Savarese et al., 2022; Soydan et al., 2023b).

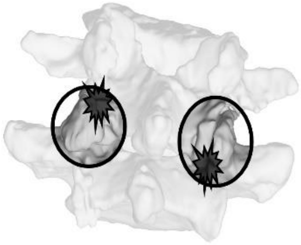

Facet joint degeneration

is associated with...

	Significantly smaller	Tendentially smaller	No difference	Tendentially larger	Significantly larger	
SS		Soydan et al., 2023a	Lv et al., 2016	Shi et al., 2024		SS
PI				Sahin et al., 2015; Soydan et al., 2023a; Muellner et al., 2022b	Jentzsch et al., 2013; Lv et al., 2016; Shi et al., 2024	PI
PT				Shi et al., 2024; Soydan et al., 2023a	Lv et al., 2016	PT
LL			Lv et al., 2016	Shi et al., 2024	Soydan et al., 2023a	LL
TK						TK
SVA						SVA

Figure 4: Literature findings on the relationship between spinopelvic alignment parameters and facet joint degeneration (Jentzsch et al., 2013; Sahin et al., 2015; Lv et al., 2016; Muellner et al., 2022b; Soydan et al., 2023a; Shi et al., 2024).

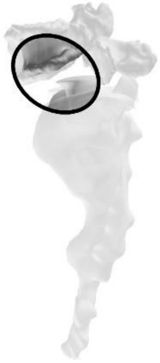

Lumbar spondylolisthesis

is associated with...

	Significantly smaller	Tendentially smaller	No difference	Tendentially larger	Significantly larger	
SS	Ferrero et al., 2015	Ha et al., 2019		Chuang et al., 2018; Chuang et al., 2021; Yin et al., 2016; Aboushaala et al., 2024; Meng et al., 2024; Oyekan et al., 2023	Funao et al., 2012; Labelle et al., 2004; Lai et al., 2018; Lim and Kim, 2014; Liu et al., 2015; Nakamae et al., 2019; Oh et al., 2013; Schuller et al., 2011; Vialle et al., 2007; Wang et al., 2016; Kong et al., 2023; Leng et al., 2023; Shrestha et al., 2021	SS
PI		Ha et al., 2019		Yin et al., 2016; Aboushaala et al., 2024; Shi et al., 2022	Barrey et al., 2007b; Chuang et al., 2018; Chuang et al., 2021; Ferrero et al., 2015; Funao et al., 2012; Labelle et al., 2004; Lai et al., 2018; Lim and Kim, 2013; Lim and Kim, 2014; Liu et al., 2015; Nakamae et al., 2019; Oh et al., 2013; Schuller et al., 2011; Vialle et al., 2007; Wang et al., 2016; Meng et al., 2024; Kong et al., 2023; Oyekan et al., 2023; Shrestha et al., 2021	PI
PT		Ha et al., 2019	Lai et al., 2018	Chuang et al., 2021; Funao et al., 2012; Lim and Kim, 2014; Liu et al., 2015; Nakamae et al., 2019; Oh et al., 2013; Yin et al., 2016; Aboushaala et al., 2024; Kong et al., 2023	Chuang et al., 2018; Ferrero et al., 2015; Labelle et al., 2004; Lim and Kim, 2013; Schuller et al., 2011; Vialle et al., 2007; Wang et al., 2016; Meng et al., 2024; Oyekan et al., 2023; Shrestha et al., 2021	PT
LL	Ferrero et al., 2015	Oh et al., 2013		Chuang et al., 2018; Ha et al., 2019; Lim and Kim, 2014; Liu et al., 2015; Yin et al., 2016; Aboushaala et al., 2024	Chuang et al., 2021; Funao et al., 2012; Labelle et al., 2004; Lai et al., 2018; Nakamae et al., 2019; Schuller et al., 2011; Vialle et al., 2007; Wang et al., 2016; Kong et al., 2023; Leng et al., 2023; Shrestha et al., 2021	LL
TK	Ferrero et al., 2015; Labelle et al., 2004; Vialle et al., 2007	Chuang et al., 2021; Lim and Kim, 2014; Wang et al., 2016		Ha et al., 2019; Nakamae et al., 2019	Funao et al., 2012; Lai et al., 2018	TK
SVA	Ha et al., 2019		Lai et al., 2018	Chuang et al., 2021; Nakamae et al., 2019	Lim and Kim, 2014; Wang et al., 2016	SVA

Figure 5: Literature findings on the relationship between spinopelvic alignment parameters and spondylolisthesis (Labelle et al., 2004; Barrey et al., 2007b; Vialle et al., 2007; Schuller et al., 2011; Funao et al., 2012; Lim and Kim, 2013, 2014; Oh et al., 2013; Ferrero et al., 2015; Liu et al., 2015; Wang et al., 2016; Yin et al., 2016; Chuang et al., 2018, 2021; Lai et al., 2018; Ha et al., 2019; Nakamae et al., 2019; Shrestha et al., 2021; Shi et al., 2022; Kong et al., 2023; Leng et al., 2023; Oyekan et al., 2023; Aboushaala et al., 2024; Meng et al., 2024).

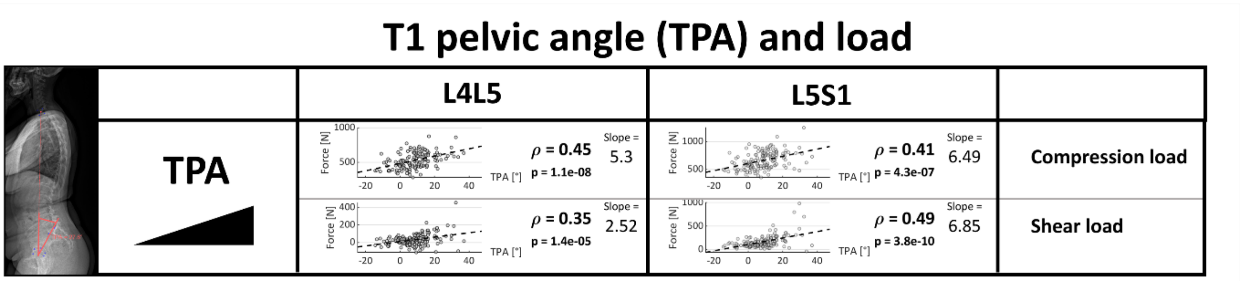

Figure 6: Strength of association between T1 pelvic angle and joint load. Significant correlations are written in bold and the slope refers to the regression line fitted on the data.

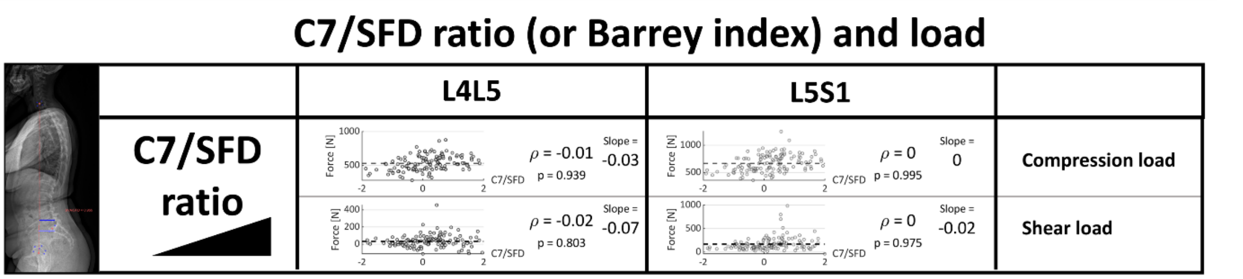

Figure 7: Strength of association between C7 to sacro-femoral distance ratio and joint load. Significant correlations are written in bold and the slope refers to the regression line fitted on the data.

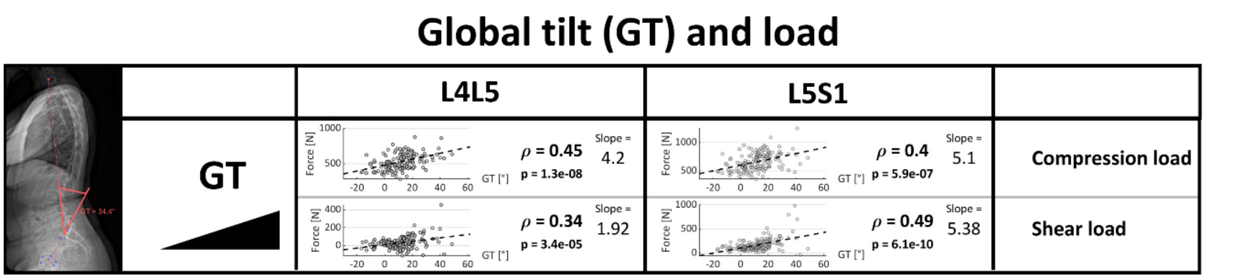

Figure 8: Strength of association between global tilt and joint load. Significant correlations are written in bold and the slope refers to the regression line fitted on the data.

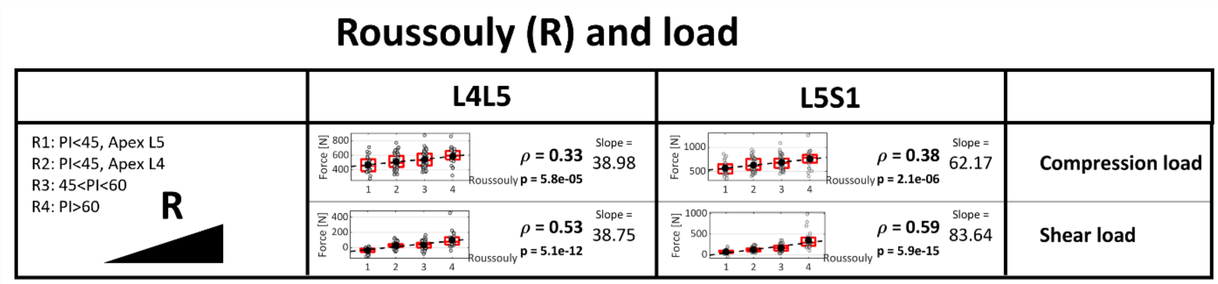

Figure 9: Strength of association between Roussouly type and joint load (Roussouly et al., 2005). Significant correlations are written in bold and the slope refers to the regression line fitted on the data.

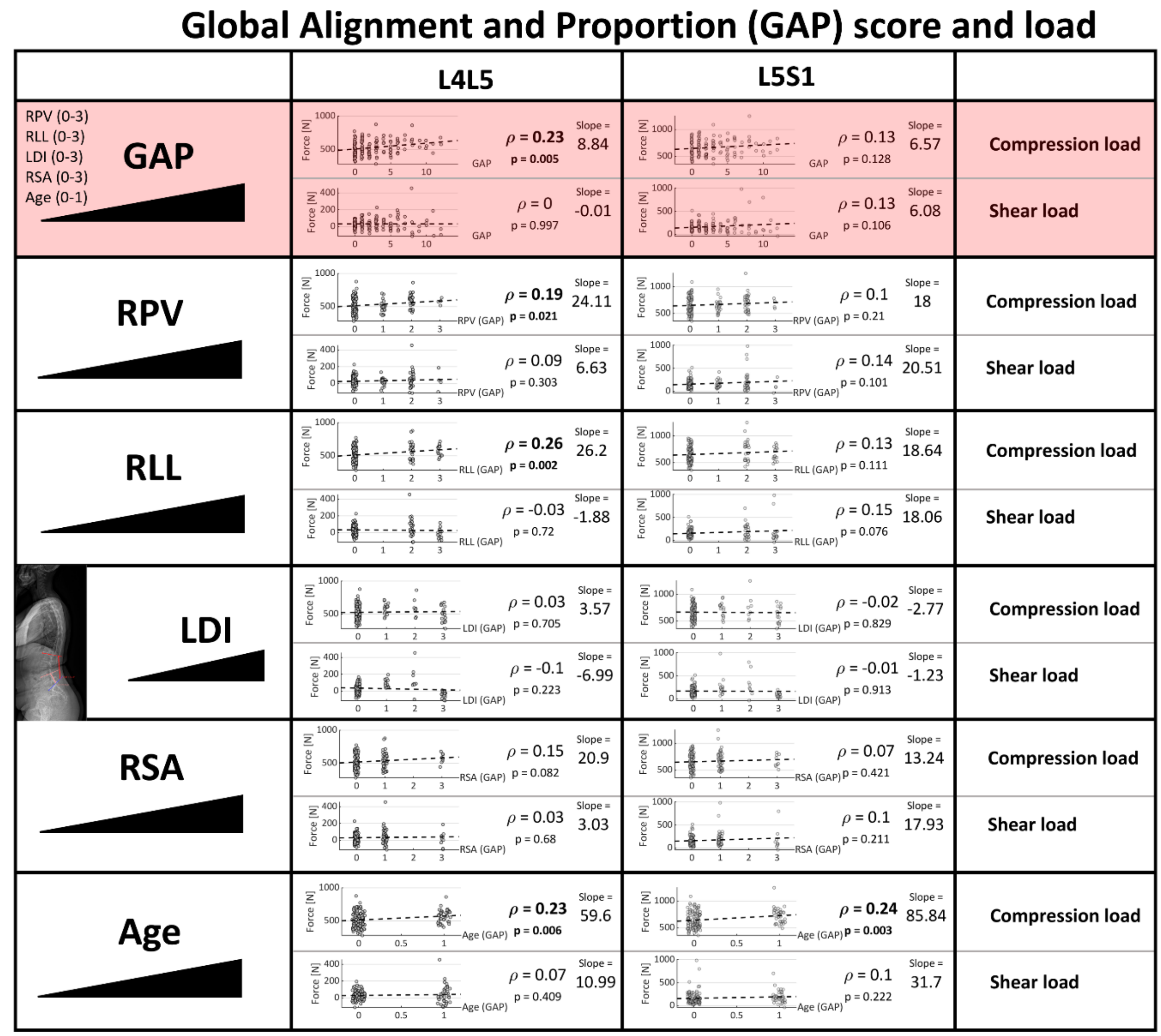

Figure 10: Strength of association between the global alignment and proportion score (and its five parameters) and joint load (Yilgor et al., 2017). Significant correlations are written in bold and the slope refers to the regression line fitted on the data. RPV: relative pelvic version; RLL: relative lumbar lordosis; LDI: lordosis distribution index; RSA: relative spinopelvic alignment.

REFERENCES

Aboushaala, K., Chee, A. V., Adnan, D., Toro, S. J., Singh, H., Savoia, A., et al. (2024). Gut microbiome dysbiosis is associated with lumbar degenerative spondylolisthesis in symptomatic patients. *JOR Spine* 7, e70005. doi: 10.1002/jsp2.70005

Bae, J., Lee, S.-H., Shin, S.-H., Seo, J. S., Kim, K. H., and Jang, J.-S. (2016). Radiological analysis of upper lumbar disc herniation and spinopelvic sagittal alignment. *Eur Spine J* 25, 1382–1388. doi: 10.1007/s00586-016-4382-y

Bao, T., Wang, C., Wang, Y., Wang, T., Zhang, Q., Gao, F., et al. (2024). Relationship between paravertebral muscle degeneration and spinal-pelvic sagittal parameters in patients with lumbar disc herniation. *Sci Rep* 14, 192. doi: 10.1038/s41598-023-50836-4

Barrey, C., Jund, J., Noseda, O., and Roussouly, P. (2007a). Sagittal balance of the pelvis-spine complex and lumbar degenerative diseases. A comparative study about 85 cases. *Eur Spine J* 16, 1459–1467. doi: 10.1007/s00586-006-0294-6

Barrey, C., Jund, J., Perrin, G., and Roussouly, P. (2007b). Spinopelvic alignment of patients with degenerative spondylolisthesis. *Neurosurgery* 61, 981–986. doi: 10.1227/01.neu.0000303194.02921.30

Chambers, A. J., Sukits, A. L., McCrory, J. L., and Cham, R. (2011). The effect of obesity and gender on body segment parameters in older adults. *Clin Biomech*, 13.

Chuang, C., Liaw, M., Wang, L., Huang, Y., Pong, Y., Chen, C., et al. (2018). Spino-pelvic alignment, balance, and functional disability in patients with low-grade degenerative lumbar spondylolisthesis. *J Rehabil Med* 50, 898–907. doi: 10.2340/16501977-2489

Chuang, H.-C., Tseng, Y.-H., Chen, Y., Chou, P.-H., Chang, W.-L., Su, P.-F., et al. (2021). Assessment of sagittal spinopelvic parameters in a Taiwanese population with spondylolysis by the EOS imaging system: a retrospective radiological analysis. *BMC Musculoskelet Disord* 22, 553. doi: 10.1186/s12891-021-04440-0

Endo, K., Suzuki, H., Tanaka, H., Kang, Y., and Yamamoto, K. (2010). Sagittal spinal alignment in patients with lumbar disc herniation. *Eur Spine J* 19, 435–438. doi: 10.1007/s00586-009-1240-1

Fasser, M.-R., Jokeit, M., Kalthoff, M., Gomez Romero, D. A., Trache, T., Snedeker, J. G., et al. (2021). Subject-Specific Alignment and Mass Distribution in Musculoskeletal Models of the Lumbar Spine. *Front. Bioeng. Biotechnol.* 9, 721042. doi: 10.3389/fbioe.2021.721042

Fei, H., Li, W., Sun, Z., Ma, Q., and Chen, Z. (2017). Analysis of Spino-pelvic Sagittal Alignment in Young Chinese Patients with Lumbar Disc Herniation. *Orthop Surg* 9, 271–276. doi: 10.1111/os.12340

Ferrero, E., Ould-Slimane, M., Gille, O., Guigui, P., and French Spine Society (SFCR) (2015). Sagittal spinopelvic alignment in 654 degenerative spondylolisthesis. *Eur Spine J* 24, 1219–1227. doi: 10.1007/s00586-015-3778-4

Funao, H., Tsuji, T., Hosogane, N., Watanabe, K., Ishii, K., Nakamura, M., et al. (2012). Comparative study of spinopelvic sagittal alignment between patients with and without degenerative spondylolisthesis. *Eur Spine J* 21, 2181–2187. doi: 10.1007/s00586-012-2374-0

Ha, J.-K., Hwang, C. J., Dong-Ho, L., Mi Young, L., So Jung, Y., and Choon Sung, L. (2019). Spinopelvic parameters in degenerative spondylolisthesis. *Acta Orthopaedica Belgica* 85, 253–259.

Jentzsch, T., Geiger, J., Bouaicha, S., Slankamenac, K., Dan Linh Nguyen-Kim, T., and Werner, C. M. (2013). Increased pelvic incidence may lead to arthritis and sagittal orientation of the facet joints at the lower lumbar spine. *BMC Med Imaging* 13, 34. doi: 10.1186/1471-2342-13-34

Kong, Q., Wei, B., Niu, S., Liao, J., Zu, Y., and Shan, T. (2023). Age, pelvic incidence, facet joint angle and pedicle-facet angle as correlative factors for isthmic spondylolisthesis: a retrospective case control study. *BMC Musculoskelet Disord* 24, 497. doi: 10.1186/s12891-023-06569-6

Labelle, H., Roussouly, P., Berthonnaud, É., Transfeldt, E., O'Brien, M., Chopin, D., et al. (2004). Spondylolisthesis, Pelvic Incidence, and Spinopelvic Balance: A Correlation Study. *Spine* 29, 2049–2054. doi: 10.1097/01.brs.0000138279.53439.cc

Lai, Q., Gao, T., Lv, X., Liu, X., Wan, Z., Dai, M., et al. (2018). Correlation between the sagittal spinopelvic alignment and degenerative lumbar spondylolisthesis: a retrospective study. *BMC Musculoskelet Disord* 19, 151. doi: 10.1186/s12891-018-2073-z

Leng, *Yebo, Tang, C., He, B., Pu, X., Kang, M., Liao, Y., et al. (2023). Correlation between the spinopelvic type and morphological characteristics of lumbar facet joints in degenerative lumbar spondylolisthesis. *Journal of Neurosurgery: Spine* 38, 425–435. doi: 10.3171/2022.11.SPINE22979

Lim, J. K., and Kim, S. M. (2013). Difference of Sagittal Spinopelvic Alignments between Degenerative Spondylolisthesis and Isthmic Spondylolisthesis. *J Korean Neurosurg Soc* 53, 96. doi: 10.3340/jkns.2013.53.2.96

Lim, J. K., and Kim, S. M. (2014). Comparison of Sagittal Spinopelvic Alignment between Lumbar Degenerative Spondylolisthesis and Degenerative Spinal Stenosis. *J Korean Neurosurg Soc* 55, 331. doi: 10.3340/jkns.2014.55.6.331

Liu, H., Li, S., Zheng, Z., Wang, J., Wang, H., and Li, X. (2015). Pelvic retroversion is the key protective mechanism of L4–5 degenerative spondylolisthesis. *Eur Spine J* 24, 1204–1211. doi: 10.1007/s00586-014-3395-7

Lv, X., Liu, Y., Zhou, S., Wang, Q., Gu, H., Fu, X., et al. (2016). Correlations between the feature of sagittal spinopelvic alignment and facet joint degeneration: a retrospective study. *BMC Musculoskelet Disord* 17, 341. doi: 10.1186/s12891-016-1193-6

Menezes-Reis, R., Bonugli, G. P., Dalto, V. F., da Silva Herrero, C. F. P., Defino, H. L. A., and Nogueira-Barbosa, M. H. (2016). Association Between Lumbar Spine Sagittal Alignment

and L4-L5 Disc Degeneration Among Asymptomatic Young Adults. *Spine* 41, E1081–E1087. doi: 10.1097/BRS.0000000000001568

Meng, D., Li, D., Guo, X., and Li, W. (2024). Evaluation of the effectiveness of the femoro-sacral posterior angle system for measuring spino-pelvic morphology in high-dysplastic developmental spondylolisthesis. *Eur Spine J* 33, 3724–3736. doi: 10.1007/s00586-024-08418-z

Muellner, M., Haffer, H., Chiapparelli, E., Dodo, Y., Tan, E. T., Shue, J., et al. (2022a). Differences in lumbar paraspinal muscle morphology in patients with sagittal malalignment undergoing posterior lumbar fusion surgery. *Eur Spine J* 31, 3109–3118. doi: 10.1007/s00586-022-07351-3

Muellner, M., Kreutzinger, V., Becker, L., Diekhoff, T., Pumberger, M., Schömig, F., et al. (2022b). Unexpected Sex Differences in the Relationship of Sacroiliac Joint and Lumbar Spine Degeneration. *Diagnostics* 12, 275. doi: 10.3390/diagnostics12020275

Nakamae, T., Nakanishi, K., Kamei, N., and Adachi, N. (2019). The correlation between sagittal spinopelvic alignment and degree of lumbar degenerative spondylolisthesis. *Journal of Orthopaedic Science* 24, 969–973. doi: 10.1016/j.jos.2019.08.021

Ogon, I., Takashima, H., Morita, T., Oshigiri, T., Terashima, Y., Yoshimoto, M., et al. (2020). Association between Spinopelvic Alignment and Lumbar Intervertebral Disc Degeneration Quantified with Magnetic Resonance Imaging T2 Mapping in Patients with Chronic Low Back Pain. *Spine Surg Relat Res* 4, 135–141. doi: 10.22603/ssrr.2019-0051

Oh, Y. M., Choi, H. Y., and Eun, J. P. (2013). The Comparison of Sagittal Spinopelvic Parameters between Young Adult Patients with L5 Spondylolysis and Age-Matched Control Group. *J Korean Neurosurg Soc* 54, 207. doi: 10.3340/jkns.2013.54.3.207

Oh, Y.-M., and Eun, J.-P. (2015). Clinical Impact of Sagittal Spinopelvic Parameters on Disc Degeneration in Young Adults. *Medicine* 94, e1833. doi: 10.1097/MD.0000000000001833

Oyekan, A., Dalton, J., Fourman, M. S., Ridolfi, D., Cluts, L., Couch, B., et al. (2023). Multilevel tandem spondylolisthesis associated with a reduced “safe zone” for a transpsoas lateral lumbar interbody fusion at L4–5. *Neurosurgical Focus* 54, E5. doi: 10.3171/2022.10.FOCUS22605

Pearsall, D. J., Reid, J. G., and Livingston, L. A. (1996). Segmental inertial parameters of the human trunk as determined from computed tomography. *Ann Biomed Eng* 24, 198–210. doi: 10.1007/BF02667349

Pourabbas Tahvildari, B., Masroori, Z., Erfani, M. A., Solooki, S., and Vosoughi, A. R. (2021). The impact of spino-pelvic parameters on pathogenesis of lumbar disc herniation. *Musculoskelet Surg* 106, 195–199. doi: 10.1007/s12306-020-00693-5

Rajnics, P., Templier, A., Skalli, W., Lavaste, F., and Illes, T. (2002). The importance of spinopelvic parameters in patients with lumbar disc lesions. *Int Orthop* 26, 104–108. doi: 10.1007/s00264-001-0317-1

Roussouly, P., Gollogly, S., Berthonnaud, E., and Dimnet, J. (2005). Classification of the Normal Variation in the Sagittal Alignment of the Human Lumbar Spine and Pelvis in the Standing Position: *Spine* 30, 346–353. doi: 10.1097/01.brs.0000152379.54463.65

Sahin, M. S., Ergün, A., and Aslan, A. (2015). The Relationship Between Osteoarthritis of the Lumbar Facet Joints and Lumbosacropelvic Morphology: *Spine* 40, E1058–E1062. doi: 10.1097/BRS.0000000000001070

Savarese, L. G., Menezes-Reis, R., Jorge, M., Salmon, C. E. G., Herrero, C. F. P. S., and Nogueira-Barbosa, M. H. (2022). Sagittal balance and intervertebral disc composition in patients with low back pain. *Braz J Med Biol Res* 55, e12015. doi: 10.1590/1414-431x2022e12015

Schuller, S., Charles, Y. P., and Steib, J.-P. (2011). Sagittal spinopelvic alignment and body mass index in patients with degenerative spondylolisthesis. *Eur Spine J* 20, 713–719. doi: 10.1007/s00586-010-1640-2

Shi, H., Li, S., Liu, S., Hu, W., Chen, J., Chen, Y., et al. (2024). Facet joint tropism, pelvic incidence and intervertebral height index: associations with facet joint osteoarthritis in lumbar spinal stenosis. *The Spine Journal* 24, 317–324. doi: 10.1016/j.spinee.2023.10.001

Shi, J., Kurra, S., Danaher, M., Bailey, F., Sullivan, K. H., and Lavelle, W. (2022). The Reliability of CT Scan Measurements of Pelvic Incidence in the Evaluation of Adult Spondylolisthesis. *Cureus*. doi: 10.7759/cureus.21696

Shrestha, S., Lakhey, R. B., Paudel, S., Kafle, D., and Pokharel, R. (2021). Correlation of Pelvic Parameters with Isthmic Spondylolisthesis. *Kathmandu Univ. Med. J.* 19, 420–423. doi: 10.3126/kumj.v19i4.49754

Song, J., Pan, F., Kong, C., Sun, X., Wang, Y., Wang, W., et al. (2022). Does the sagittal spinal profile differ between the elderly Chinese populations with and without lumbar disc herniation? *Asian Journal of Surgery* 45, 2719–2724. doi: 10.1016/j.asjsur.2022.03.020

Soydan, Z., Bayramoglu, E., and Altas, O. (2023a). The Impact of Spinopelvic Alignment on the Facet Joint Degeneration. *Global Spine Journal*, 21925682231162813. doi: 10.1177/21925682231162813

Soydan, Z., Bayramoglu, E., and Sen, C. (2023b). Elucidation of effect of spinopelvic parameters in degenerative disc disease. *Neurochirurgie* 69, 101388. doi: 10.1016/j.neuchi.2022.101388

Vialle, R., Ilharreborde, B., Dauzac, C., Lenoir, T., Rillardon, L., and Guigui, P. (2007). Is there a sagittal imbalance of the spine in isthmic spondylolisthesis? A correlation study. *Eur Spine J* 16, 1641–1649. doi: 10.1007/s00586-007-0348-4

Wang, T., Ma, L., Yang, D.-L., Wang, H., Zhang, D., Zhang, Y.-Z., et al. (2017). Radiological analysis for thoracolumbar disc herniation in spinopelvic sagittal alignment: A retrospective study. *Medicine* 96, e6593. doi: 10.1097/MD.0000000000006593

Wang, T., Wang, H., Liu, H., Ma, L., Liu, F.-Y., and Ding, W.-Y. (2016). Sagittal spinopelvic parameters in 2-level lumbar degenerative spondylolisthesis: A retrospective study. *Medicine* 95, e5417. doi: 10.1097/MD.0000000000005417

Wu, W., Chen, Y., Yu, L., Li, F., and Guo, W. (2019). Coronal and sagittal spinal alignment in lumbar disc herniation with scoliosis and trunk shift. *J Orthop Surg Res* 14, 264. doi: 10.1186/s13018-019-1300-0

Wu, Z.-M., Ji, X.-Q., Lian, K., and Liu, J.-T. (2020). Analysis of the Relationship Between Modic Change and Spinopelvic Parameters in the Sagittal Plane. *Med Sci Monit* 26. doi: 10.12659/MSM.919667

Yang, X., Kong, Q., Song, Y., Liu, L., Zeng, J., and Xing, R. (2014). The characteristics of spinopelvic sagittal alignment in patients with lumbar disc degenerative diseases. *Eur Spine J* 23, 569–575. doi: 10.1007/s00586-013-3067-z

Yilgor, C., Sogunmez, N., Boissiere, L., Yavuz, Y., Obeid, I., Kleinstück, F., et al. (2017). Global Alignment and Proportion (GAP) Score: Development and Validation of a New Method of Analyzing Spinopelvic Alignment to Predict Mechanical Complications After Adult Spinal Deformity Surgery. *The Journal of Bone and Joint Surgery* 99, 1661–1672. doi: 10.2106/JBJS.16.01594

Yin, J., Peng, B.-G., Li, Y.-C., Zhang, N.-Y., Yang, L., and Li, D.-M. (2016). Differences of Sagittal Lumbosacral Parameters between Patients with Lumbar Spondylolysis and Normal Adults. *Chinese Medical Journal* 129, 1166–1170. doi: 10.4103/0366-6999.181972

Zehra, U., Cheung, J. P. Y., Bow, C., Crawford, R. J., Luk, K. D. K., Lu, W., et al. (2020). Spinopelvic alignment predicts disc calcification, displacement, and Modic changes: Evidence of an evolutionary etiology for clinically-relevant spinal phenotypes. *JOR Spine* 3. doi: 10.1002/jsp2.1083
